# KDM8 epigenetically controls cardiac metabolism to prevent initiation of dilated cardiomyopathy

**DOI:** 10.1038/s44161-023-00214-0

**Published:** 2023-02-13

**Authors:** Abdalla Ahmed, Jibran Nehal Syed, Lijun Chi, Yaxu Wang, Carmina Perez-Romero, Dorothy Lee, Etri Kocaqi, Amalia Caballero, Jielin Yang, Quetzalcoatl Escalante-Covarrubias, Akihiko Ishimura, Takeshi Suzuki, Lorena Aguilar-Arnal, Gerard Bryan Gonzales, Kyoung-Han Kim, Paul Delgado-Olguín

**Affiliations:** 1 Department of Translational Medicine, The Hospital for Sick Children, Toronto, Ontario Canada; 2 Department of Molecular Genetics, University of Toronto, Toronto, Ontario Canada; 3 Department of Physiology, University of Toronto, Toronto, Ontario Canada; 4 Departamento de Biología Celular y Fisiología, Instituto de Investigaciones Biomédicas, Universidad Nacional Autónoma de México, México City, México; 5 Division of Functional Genomics, Cancer Research Institute, Kanazawa University, Kanazawa, Japan; 6 Division of Human Nutrition and Health, Wageningen University, Wageningen, Netherlands; 7 Department of Cellular and Molecular Medicine, Faculty of Medicine, University of Ottawa and University of Ottawa Heart Institute, Ottawa, Ontario Canada; 8 Heart & Stroke Richard Lewar Centre of Excellence, Toronto, Ontario Canada

**Keywords:** Histone post-translational modifications, Heart failure, Metabolism, Gene expression analysis

## Abstract

Cardiac metabolism is deranged in heart failure, but underlying mechanisms remain unclear. Here, we show that lysine demethylase 8 (Kdm8) maintains an active mitochondrial gene network by repressing *Tbx15*, thus preventing dilated cardiomyopathy leading to lethal heart failure. Deletion of *Kdm8* in mouse cardiomyocytes increased H3K36me2 with activation of *Tbx15* and repression of target genes in the NAD^+^ pathway before dilated cardiomyopathy initiated. NAD^+^ supplementation prevented dilated cardiomyopathy in *Kdm8* mutant mice, and *TBX15* overexpression blunted NAD^+^-activated cardiomyocyte respiration. Furthermore, *KDM8* was downregulated in human hearts affected by dilated cardiomyopathy, and higher *TBX15* expression defines a subgroup of affected hearts with the strongest downregulation of genes encoding mitochondrial proteins. Thus, KDM8 represses *TBX15* to maintain cardiac metabolism. Our results suggest that epigenetic dysregulation of metabolic gene networks initiates myocardium deterioration toward heart failure and could underlie heterogeneity of dilated cardiomyopathy.

## Abstract (web summary)

Ahmed et al. show that lysine demethylase 8 (Kdm8) maintains the homeostatic cardiac metabolism by repressing *Tbx15*, thus preventing dilated cardiomyopathy in mice. In humans, *KDM8* was downregulated in hearts affected by dilated cardiomyopathy, and higher *TBX15* expression correlated with the strongest downregulation of genes encoding mitochondrial proteins.

## Main

Dilated cardiomyopathy (DCM) is characterized by an enlarged and poorly contractile left ventricle that does not eject enough blood to meet the body’s need for nutrients and oxygen. As such, DCM is a major cause of heart failure^1^, and the main indicator for heart transplantation^2^. The myocardium adversely remodels at end-stage DCM^3^ with thinning of the ventricular myocardium causing dilation of the ventricles, which subjects the myocardium to increased mechanical stress. This promotes cardiomyocyte death^4^ and extracellular matrix remodeling leading to increased fibrosis^5^. Moreover, mitochondria morphology, number and distribution^6–8^ are altered, and mitochondrial oxidative capacity is decreased in DCM^9–11^. Decreased oxidation of fatty acids depletes ATP, altering energy metabolism and decreasing the heart’s contractile efficiency^12,13^. Heart failure is strongly associated with inborn errors of metabolism^14^ and gene mutations affecting oxidative metabolism^15–17^, which decreases proportionally to DCM severity^18^. Moreover, activating the biosynthesis or salvage of nicotine adenine dinucleotide (NAD^+^), a key coenzyme in redox reactions and mitochondrial ATP generation, preserves heart function in DCM mouse models^19,20^. This suggests that cardiac metabolism alterations could cause and regulate DCM progression. However, the cause of DCM is known only for the ~30% of people affected by inherited forms of the disease. For the remaining ~70%, the cause is unknown, and consensus has not yet been reached on whether altered metabolism causes, or is secondary to, adverse myocardial remodeling in DCM^21^.

Histone methyltransferases and demethylases are epigenetic regulators that use metabolites as substrates to deposit or remove methyl groups on histones and other proteins in response to, or to influence, transcription^22^. The function of histone methyltransferases and demethylases in development and disease is still poorly understood; however, they are emerging as regulators of cardiac homeostasis and heart failure^23–25^. Histone methyl marks associated with active and inactive transcription are abnormally distributed across the genome in the human failing heart^26,27^. The lysine demethylase 8, or Kdm8, demethylates the di-methylated form of lysine 36 of the histone H3 (H3K36me2). H3K36me2 is mediated by NSD1-3, ASH1L, SMYD2, SETMAR and SETD3 and is predominantly associated with active transcription^28^. However, some evidence suggests a function in gene repression. H3K36me2 is enriched in the body of transcriptionally active genes and limits the spread of H3K27me3 (ref. ^29^), which associates predominantly with repressed chromatin. In contrast, decreased H3K36me2 following NSD1 knockdown tracks with enhancer activation and expression of drivers of embryonic stem cell differentiation^30^. Moreover, H3K36me2 at gene promoters in yeast cross talks with histone deacetylases, suggesting a function of H3K36me2 in transcriptional repression^31^. KDM8 controls metabolic programming in cancer cells^32,33^, it is strongly expressed in the embryonic mouse heart^34^, and *Kdm8* knockout mouse embryos die with pericardial edema^35^, suggestive of embryonic heart failure. Moreover, *KDM8* missense variants have been associated with Coffin–Siris syndrome (ClinVar; VCV000986392.1), which presents with congenital heart defects^36^. However, the function of KDM8 in cardiomyocytes, cardiac maintenance or disease is unknown.

Histone modifiers control cardiac-specific gene expression and cardiac homeostasis through key transcription factors^37^. T-box factors (Tbx) are a family of transcriptional activators and repressors that compete to bind different combinations of half T sites as heterodimers to control multiple developmental and physiological processes^38,39^. Tbx15 is a transcriptional repressor predominantly expressed in the mouse developing limb bud and embryonic mesenchymal condensations of forming endochondral bone. Accordingly, skeletal development is abnormal in *Tbx15* mutant embryos^40^. Growing evidence suggests that Tbx15 is a metabolic master regulator. Pre-adipocytes derived from fibroblasts overexpressing *Tbx15* have impaired differentiation, a lower mitochondrial mass, and decreased respiratory capacity^41^. Moreover, Tbx15 is required for adipocyte browning^42^, and TBX15-controlled networks explain polygenic risk for abdominal obesity and diabetes in humans^43^. In skeletal muscle, Tbx15 inactivation increases development of glycolytic fibers while decreasing oxidative fibers^44^. Intriguingly, *Tbx15* is overexpressed in cardiomyocytes of mice mutant for phospholamban that develop DCM, but not in mutants for the myosin heavy chain gene that develop hypertrophic cardiomyopathy that does not progress to heart failure^45^. This suggests that Tbx15 might derange cardiac metabolism when overexpressed in the failing heart. However, the mechanisms controlling expression of *Tbx15* or its function in the heart are unknown.

## Results

### Loss of Kdm8 causes DCM leading to heart failure

**Fig. 1:**
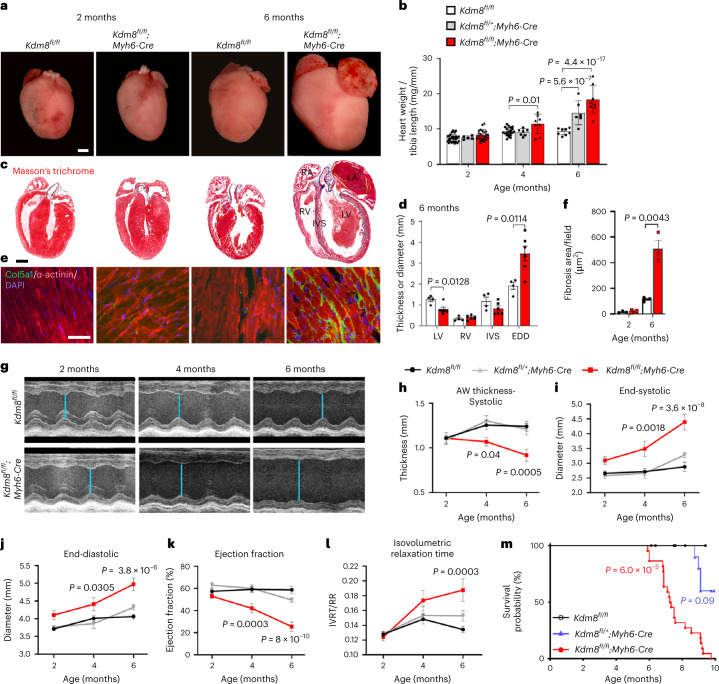
Cardiomyocyte-specific loss of Kdm8 causes DCM leading to lethal heart failure. **a**, Hearts from 2-month-old and 6-month-old *Kdm8*^*fl/fl*^ control and *Kdm8*^*fl/fl*^*;Myh6-Cre* mutant mice. Scale bar, 1 mm. **b**, Heart weight normalized to the tibia length of control, heterozygous (*Kdm8*^*fl/+*^*;Myh6-Cre*), and mutant mice. Data are mean ± s.e.m. Two-way ANOVA with multiple comparison and Sidak correction. *n* = 27 control, 5 heterozygous, and 25 mutant 2-month-old mice per group. *n* = 21 control, 7 heterozygous, and 8 mutant 4-month-old mice. *n* = 8 control, 6 heterozygous, and 8 mutant 6-month-old mice. **c**, Representative sections of three hearts from 2-month-old and 6-month-old control and mutant mice, stained with Masson’s trichrome. RA, right atrium; LA, left atrium; RV, right ventricle; LV, left ventricle; IVS, interventricular septum. Scale bar, 1 mm. **d**, Thickness of the wall of the LV, RV, and IVS, and diameter of the LV at end-diastole (EDD) measured from sections of 6-month-old hearts. Data are mean ± s.e.m. Two-tailed Student’s *t*-test. *n* = 4 controls and 6 mutants. **e**, Immunofluorescence of Col5a1 and α-actinin on sections of 2-month-old and 6-month-old hearts. Nuclei were counterstained with 4′,6-diamidino-2-phenylindole (DAPI). Scale bar, 50 μm. Images are representative of three independent staining experiments. **f**, Area of Col5a1 signal in view field. Data are mean ± s.e.m. Two-tailed Student’s *t*-test. *n* = 3 mice per group. **g**, Echocardiogram of the LV of control and mutant mice. The blue line shows the diameter of the LV at end-systole. **h**, LV anterior wall (AW) thickness at end-systole. **i**, LV end-systolic diameter. **j**, LV end-diastolic diameter. **k**, LV ejection fraction. **l**, Isovolumetric relaxation time normalized to the time between heartbeats (IVRT/RR). Data in **h** to **l** are mean ± s.e.m. Two-way ANOVA with multiple comparison correction by Tukey’s method. *n* = 6–11 mice per group (see Source data for *P* values). **m**, Survival curve of control, *Kdm8* heterozygous, and mutant mice analyzed by log-rank (Mantel–Cox) test. *n* = 10 control, 10 heterozygous, and 21 mutant mice. Source data

**Extended Data Fig. 1:**
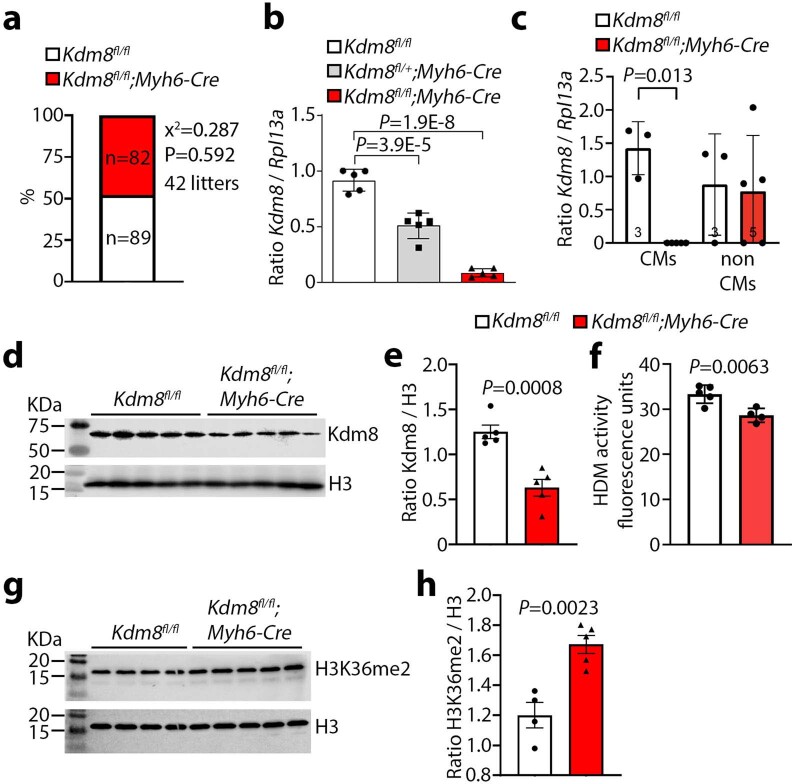
Kdm8 demethylates H3K36me2 in the heart. **a**, Percentage of genotypes of male offspring from control *Kdm8*^*fl/fl*^ females crossed with mutant *Kdm8*^*fl/fl*^*;Myh6-Cre* males. Data was analyzed by two-tailed chi-squared test. **b**, qPCR of *Kdm8* normalized to *Rpl13a* in whole ventricles of 2-month-old control, heterozygous (*Kdm8*^*fl+*^*;Myh6-Cre*), and mutant mice. Error bars denote the mean + /- s.d. Data was analyzed by one-way ANOVA with Tuckey’s multiple comparison correction. n = 5 hearts per group. **c**, qPCR of *Kdm8* in isolated cardiomyocytes (CMs) and in the non-cardiomyocyte cell fraction (non CMs) of 2-month-old control and mutant hearts. Error bars denote the mean + /- s.d. Data was analyzed by two-way ANOVA. n = 3 control and 5 mutant hearts. qPCR in **b** and **c** were repeated twice with similar results. **d**, Western blot of Kdm8 in whole ventricles at 6 months of age. **e**, Band intensity of Kdm8 protein normalized to histone H3 (H3) (shown in **d**) and analyzed by unpaired two-tailed Student’s t-test. n = 5 hearts per group. Error bars denote the mean + /- s.e.m. **f**, Histone demethylase (HDM) activity of Jumonji domain-containing proteins, shown as fluorescence units, on extracts of control and mutant hearts at 5 weeks of age. Error bars denote the mean + /- s.d. Data was analyzed by unpaired two-tailed Student’s t-test. n = 5 control and 4 mutant hearts. **g**, Western blot of H3K36me2 in whole ventricles at 2 months of age. **h**, Band intensity of H3K36me2 normalized to H3 (shown in **g**) and analyzed by two-tailed Student’s t-test. error bars denote the mean + /- s.e.m. n = 5 control and 5 mutant hearts. Source data

**Extended Data Fig. 2:**
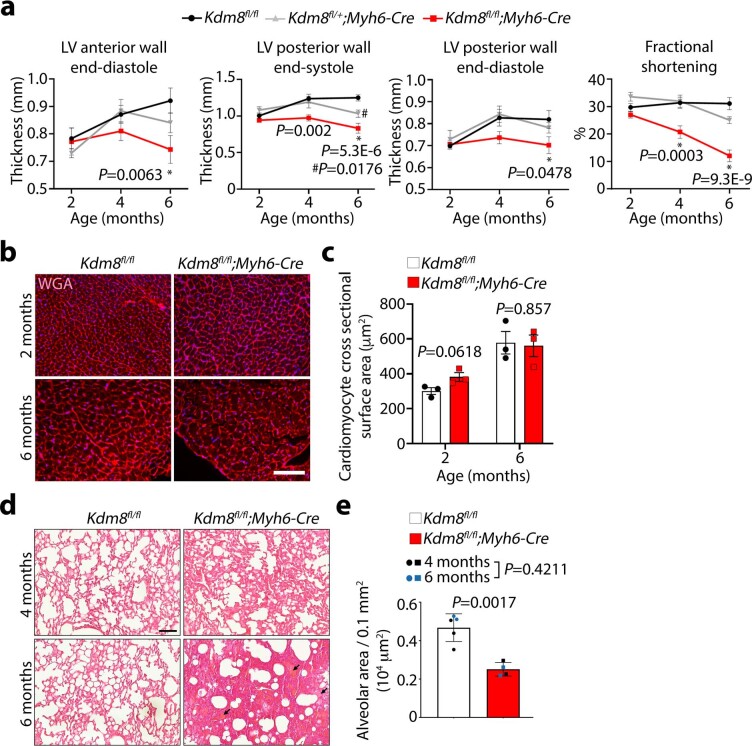
*Kdm8* mutants develop dilated cardiomyopathy with no signs of concentric hypertrophy. **a**, Echocardiogram analysis of left ventricle (LV) anterior wall thickness at end-diastole, LV posterior wall thickness at end-systole, LV posterior wall thickness at end-diastole, and LV fractional shortening in control (*Kdm8*^*fl/fl*^), heterozygous (*Kdm8*^*fl/+*^*;Myh6-Cre*), and mutant (*Kdm8*^*fl/fl*^*;Myh6-Cre*) mice at 2, 4 and 6 months of age. Error bars denote the mean + /- s.e.m. Data was analyzed by 2-way ANOVA with Tuckey’s multiple comparison correction. * denotes comparisons between control and mutants, # between control and heterozygous hearts. n = 6 - 11 mice per group. **b**, Wheat germ agglutinin staining (WGA) on cross sections of the hearts of 2 and 6-month-old control and mutant mice. Scale bar, 100 μm. **c**, Cell surface area quantified from sections stained with WGA and analyzed by two-tailed Student’s t-test. Error bars denote the mean + /- s.e.m. n = 3 hearts per group. **d**, Lung sections of control and *Kdm8* mutants at 4 and 6 months of age stained with H&E. Arrows point to areas of accumulated alveolar fluid. Scale bar = 100 μm. **e**, Alveolar area per 0.1 mm^2^ in lung sections Error bars denote the mean + /- s.d. Data was analyzed by two-way ANOVA with Sidak correction. n = 3 4-month-old and 2 6-month-old lungs of controls, and 2 4-month-old and 2 6-month-old lungs of mutant mice.

To determine the function of Kdm8 in the heart, we mutated its gene in mouse embryonic cardiomyocytes by Cre-mediated recombination. Mice with *loxP* sites flanking the catalytic domain-encoding region of *Kdm8* (*Kdm8*^*fl/fl*^)^35^ were crossed with mice expressing Cre recombinase driven by the α-myosin heavy chain (*Myh6*) promoter^46^. Homozygous *Kdm8*^*fl/fl*^*;Myh6-Cre* mice were viable, fertile and born in Mendelian ratios (Extended Data Fig. 1a). Kdm8 was efficiently inactivated; *Kdm8* mRNA was decreased by 2-fold in heterozygous *Kdm8*^*fl/+*^*;Myh6-Cre* and 10-fold in homozygous mutant hearts compared with *Kdm8*^*fl/fl*^ controls (Extended Data Fig. 1b). *Kdm8* mRNA level was not affected in non-cardiomyocytes of *Kdm8* mutant hearts, but it was ablated in cardiomyocytes (Extended Data Fig. 1c). Kdm8 protein, and histone demethylase activity were decreased (Extended Data Fig. 1d–f), while histone H3 di-methylated at lysine 36 (H3K36me2) globally increased in *Kdm8* mutant hearts (Extended Data Fig. 1g,h). Heart mass was normal in *Kdm8* mutants at 2 months of age; however, it progressively enlarged at 4 and 6 months (Fig. 1a,b). Histological analysis revealed a thinner left ventricle wall and a wider left ventricle end-diastolic diameter at 6 months (Fig. 1c,d). However, the thickness of the right ventricular wall was normal (Fig. 1c,d). The interventricular septum thickness was variable but tended to decrease (Fig. 1c,d), perhaps reflecting slightly different stages of progressing DCM in 6-month-old mutants. Interstitial fibrosis was comparable between control and mutant hearts at 2 months; however, it increased significantly in mutants at 6 months (Fig. 1e,f). Echocardiography confirmed that the left ventricle wall thickness was normal at 2 months, but progressively decreased at 4 and 6 months (Fig. 1g,h and Extended Data Fig. 2a). Similarly, the left ventricle diameter was normal in *Kdm8* mutants at 2 months, but progressively increased at 4 and 6 months (Fig. 1g,i,j). Systolic function indicated by ejection fraction and fractional shortening, and diastolic function indicated by the isovolumetric relaxation time, were normal at 2 months (Fig. 1k,l and Extended Data Fig. 2a). In contrast, systolic function was reduced at 4 and 6 months (Fig. 1g,k), while diastolic function was reduced at 6 months of age (Fig. 1l). These results indicate that Kdm8 deficiency in cardiomyocytes causes DCM that onsets at 2 months and progresses at 4 and 6 months of age. DCM was not preceded by concentric hypertrophy because cardiomyocyte cross-sectional area was normal in *Kdm8* mutants at 2 and 6 months (Extended Data Fig. 2b,c). *Kdm8* mutants had reduced alveolar space at 4 months of age, and accumulated alveolar fluid at 6 months, indicating pulmonary edema (Extended Data Fig. 2d,e). Mutants reached endpoint due to heart failure at a median age of 7.3 months (Fig. 1m). Although cardiac function was less affected in heterozygous mutants (Fig. 1i,k,l), they reached endpoint at a median age of 9.5 months (Fig. 1m), indicating that *Kdm8* haploinsufficiency causes a less severe DCM than that caused by total Kdm8 depletion. This suggests that basal cardiac homeostasis is sensitive to decreased Kdm8 abundance in cardiomyocytes.

Prolonged Cre overexpression can have toxic effects in cardiomyocytes^47^. We addressed potential cardiotoxicity of Myh6-Cre by using it to delete *L3mbtl2*, which encodes a subunit of the polycomb repressive complex 1. This did not cause DCM or premature lethality in mutants by 22 months of age (data not shown). Moreover, cardiac morphology and contractile function were normal in 6-month-old *Myh6-Cre* mice (Supplementary Fig. 1). Despite the fact that these controls do not fully rule out a potential effect of Cre as a DCM modifier, they suggest minimal or no contribution of Myh6-Cre-mediated toxicity to DCM in *Kdm8* mutants at 6 months of age.

**Extended Data Fig. 3:**
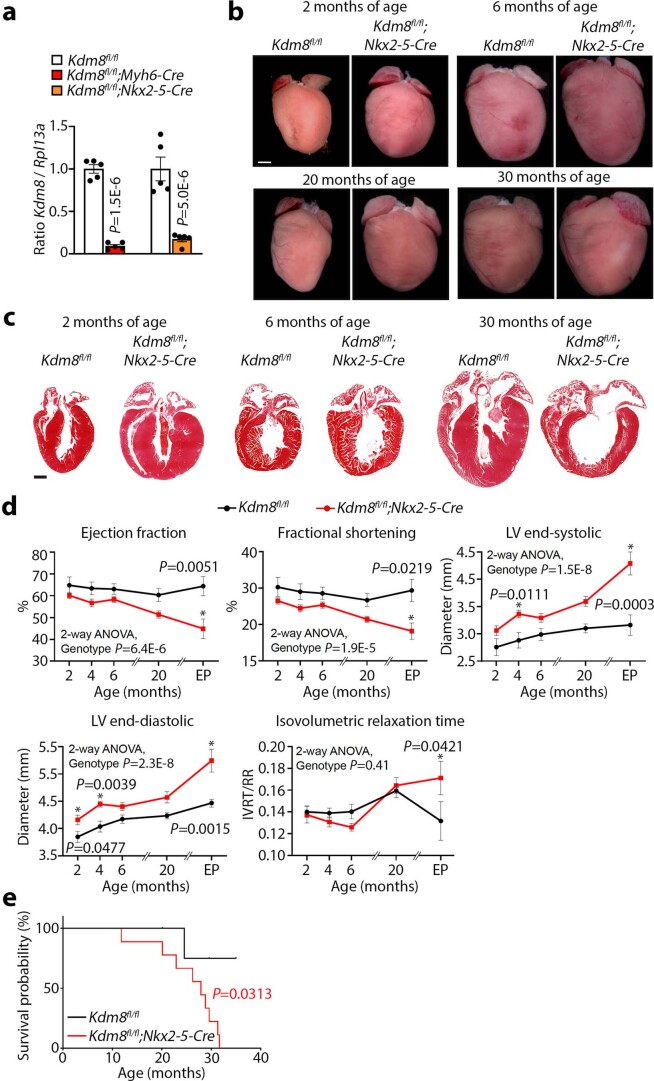
Loss of Kdm8 in cardiac progenitor cells causes dilated cardiomyopathy. **a**, qPCR of *Kdm8* normalized to *Rpl13a* on 2-month-old control (*Kdm8*^*fl/fl*^) and mutant (*Kdm8*^*fl/fl*^*;Myh6-Cre* or *Kdm8*^*fl/fl*^*;Nkx2-5-Cre*) hearts. Error bars denote the mean + /- s.e.m. Data was analyzed by two-way ANOVA. n = 5 hearts per group. The experiment was repeated twice with similar results. **b**, Hearts of control and mutant mice at 2, 6, 20, and 30 months of age. Scale bar, 1 mm. **c**, Sections of 2, 6 and 30-month-old control and mutant hearts stained with H&E. Scale bar, 1 mm. **d**, Echocardiogram analysis of left ventricle (LV) ejection fraction, fractional shortening, diameter at end-systole and end-diastole, and isovolumetric relaxation time normalized to the time between heartbeats (IVRT/RR) in control and mutant mice. Error bars denote the mean + /- s.e.m. Data was analyzed by 2-way ANOVA with Sidak’s multiple comparison correction. n = 10 – 11 mice at 2, 4 and 6 months, n = 5 controls and 7 mutants at 20 months, and n = 3 controls and 4 mutants at end point (EP) (see Supplementary Table 2 for *P* values). * *P* < 0.05, ** *P* < 0.01, *** *P* < 0.001. **e**, Survival curve of control, and mutant mice analyzed by Log-rank (Mantel-Cox) test. n = 5 control mice and 9 mutants. Source data

*Kdm8* deletion in a broader population of cardiac progenitors using Nkx2-5-Cre^48^ (Extended Data Fig. 3a) caused DCM that progressed beyond 20 months of age (Extended Data Fig. 3b–d), perhaps because of less efficient recombination in cardiomyocytes (Extended Data Fig. 3a)^48^, or non-cardiomyocyte-mediated compensatory effects. Nonetheless, *Kdm8*^*fl/fl*^*;Nkx2-5-Cre* mutant mice reached endpoint at a median age of 27.9 months. At this age, the survival rate of control mice was 75%, whereas that of mutants significantly decreased to 44% (Extended Data Fig. 3e), indicating premature lethality associated with DCM.

Thus, Kdm8 is an H3K36me2 demethylase in cardiomyocytes that is required for cardiac homeostasis, and its deficiency causes progressive DCM and premature lethality due to heart failure.

### Kdm8 maintains an active mitochondrial gene network in the heart

**Fig. 2:**
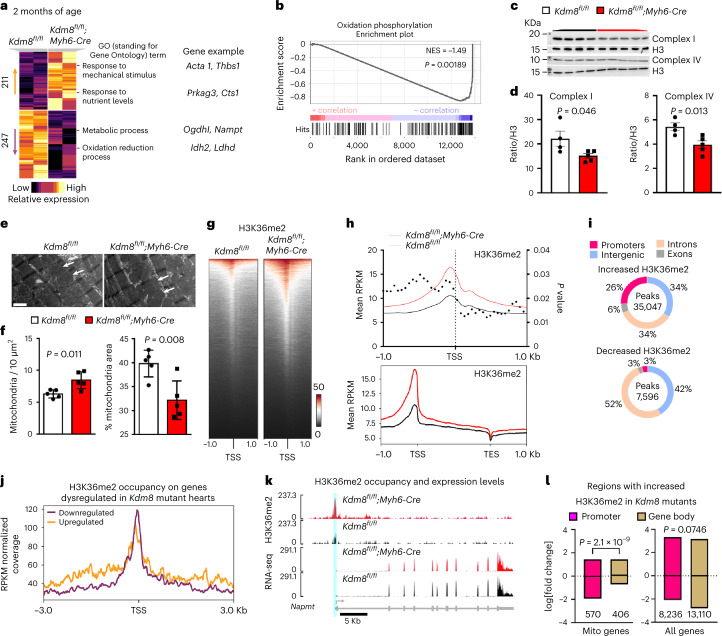
Genes regulating mitochondrial oxidative metabolism are repressed before cardiac functional decline in *Kdm8* mutant hearts. **a**, Heatmap clustering of genes differentially expressed in ventricles of 2-month-old *Kdm8*^*fl/fl*^*;Myh6-Cre* mutant vs *Kdm8*^*fl/fl*^ control mice. Enriched GO terms and representative genes are shown. **b**, GSEA including all expressed genes. *P* values were obtained from GSEA. NES, normalized enrichment score. **c**, Western blot of electron transport chain complexes I (Ndufb8) and IV (Cytochrome C) on 2-month-old ventricles. **d**, Protein relative to histone H3 in **c**. Data are mean ± s.e.m. Two-tailed Student’s *t*-test. *n* = 4 control and 5 mutant hearts. **e**, Transmission electron micrographs of the left ventricle of 2-month-old controls and mutants. Arrowheads indicate mitochondria. Scale bar, 1 μm. **f**, Mitochondria per 10 μm^2^, and percentage area occupied by mitochondria per micrograph. Data are mean ± s.d. Two-tailed Student’s *t*-test. *n* = 5 mice per group. **g**, Heatmap of H3K36me2 (red) occupancy (normalized counts) in 2-month-old control and mutant hearts 1 kb upstream and downstream of the transcription start site (TSS). **h**, H3K36me2 enrichment as reads per kilobase per million mapped reads (RPKM) 1 kb upstream and downstream of the TSS in 2-month-old control and mutant hearts. TES, transcription end site. Dots indicate *P* values. **i**, Peaks with increased or decreased H3K36me2 across promoters, introns, exons and intergenic regions in 2-month-old *Kdm8* mutant hearts. **j**, H3K36me2 occupancy RPKM 3 kb upstream and downstream of the TSS of genes that were downregulated or upregulated in 2-month-old *Kdm8* mutant hearts. **k**, H3K36me2 CUT&Tag and RNA-seq tracks of *Nampt* in 2-month-old control and mutant hearts. Units for CUT&Tag are RPKM and reads per million mapped reads (RPM) for RNA-seq. **l**, Boxplot showing the mean and range (minimum to maximum) log fold change expression of genes encoding proteins that localize to the mitochondria (sourced from MitoCarta3.0), and of all genes with increased H3K36me2 at promoters or gene bodies. Unpaired two-tailed Student’s *t*-test. The number of genes in each compared category is indicated below each bar. Source data

**Extended Data Fig. 4:**
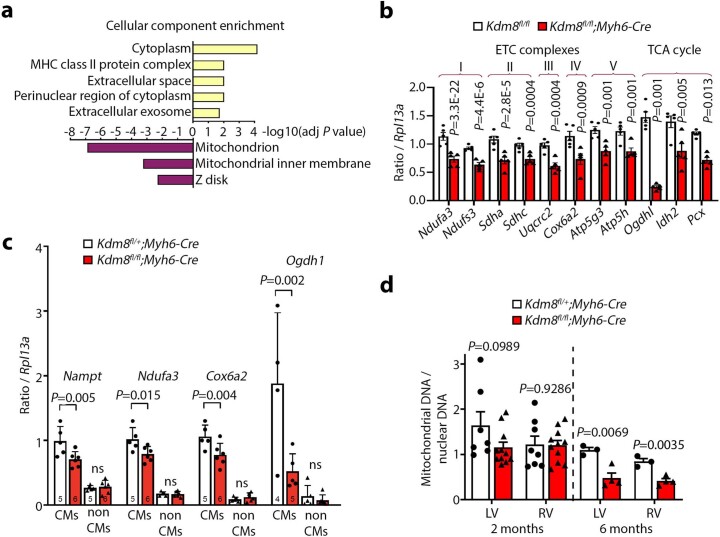
Mitochondrial function regulators are downregulated at DCM onset, and mitochondrial DNA is decreased at end-stage DCM in *Kdm8* mutant hearts. **a**, Gene ontology terms (cellular component) enriched amongst genes upregulated (yellow) and downregulated (purple) in ventricles of 2-month-old *Kdm8* mutant hearts. Data was analyzed using DAVID v6.8 with Benjamini correction. **b**, qPCR of transcripts of components of electron transport (ETC) complexes and the tricarboxylic acids (TCA) cycle on ventricles of 2-month-old control (*Kdm8*^*fl/fl*^) and mutant (*Kdm8*^*fl/fl*^*;Myh6-*Cre) hearts, analyzed by two-tailed Student’s t-test with Hold-Sidak multiple comparison correction. n = 5 mice per group. Error bars denote the mean + /- s.e.m. **c**, qPCR on cardiomyocytes (CMs) and noncardiomyocytes (non CMs) isolated from control and mutant mice at 2 months of age. Error bars denote the mean + /- s.e.m. Data was analyzed by two-way ANOVA. n for *Nampt, Ndufa3, and Cox6a2* = 5 control and 6 mutant hearts. n for *Ogdh1* = 4 control and 5 mutant hearts. **d**, qPCR of mitochondrial DNA (mtDNA) normalized to the β-globin gene as representative of nuclear DNA, on the left (LV) and right ventricle (RV) of *Kdm8*^*fl/fl*^ control and *Kdm8*^*fl/fl*^*;Myh6-Cre* mutant hearts, analyzed by two-tailed Student’s t-test. Error bars denote the mean + /- s.e.m. n = 3 to 11 mice per group. qPCR experiments were repeated twice with similar results.

To uncover the events driving initiation of DCM, we analyzed the transcriptome of ventricles at disease onset in 2-month-old *Kdm8* mutant hearts using RNA sequenceing (RNA-seq). A total of 458 genes were significantly dysregulated, of which 211 (46%) were upregulated and 247 (54%) were downregulated (Fig. 2a and Supplementary Table 1). Upregulated genes enriched for gene ontologies related to mechanical stimulation, nutrient stress, and starvation (Fig. 2a). Downregulated genes encode proteins that largely localize to the mitochondria and are associated with regulation of metabolism and oxidation–reduction (Fig. 2a and Extended Data Fig. 4a). Gene set enrichment analysis (GSEA) identified oxidative phosphorylation as the only underrepresented gene set (false discovery rate (FDR) < 25%, *P* < 1%) in *Kdm8* mutant hearts (Fig. 2b). No gene sets were overrepresented. Quantitative real-time PCR (qPCR) and western blot confirmed downregulation of enzymes in the tricarboxylic acid cycle and the electron transport chain (Fig. 2c,d and Extended Data Fig. 4b). These gene expression changes were specific to cardiomyocytes (Extended Data Fig. 4c). Moreover, transmission electron microscopy revealed that mitochondria in cardiomyocytes of the left ventricle of 2-month-old *Kdm8* mutants were more numerous and smaller than in control mice (Fig. 2e,f). Mitochondrial DNA content was not altered in *Kdm8* mutant ventricles at 2 months but declined as the mice aged (Extended Data Fig. 4d), suggesting that mitochondrial function and dynamics, but not overall mitochondrial content, are altered before morphological and functional cardiac deterioration at DCM onset.

### Kdm8 preferentially demethylates promoters of genes controlling metabolism and maintains their expression in the heart

H3K36me2 was globally increased in *Kdm8* mutant hearts (Extended Data Fig. 1g,h). We assessed the function of the histone demethylase activity of Kdm8 by mapping H3K36me2 genome wide in cardiomyocytes isolated from 2-month-old control and *Kdm8* mutant hearts. CUT&Tag revealed 12,457 more H3K36me2 peaks in mutant (81,807 peaks) vs control (69,350 peaks) cardiomyocytes. Consistent with loss of demethylase activity in *Kdm8* mutant hearts, 35,047 genomic regions had increased levels of H3K36me2 (Fig. 2g), and only 7,596 had decreased levels. H3K36me2 occupancy was most significantly increased in the gene body immediately downstream of the transcription start site (Fig. 2h). Genomic regions 2.5 kilobases (kb) upstream or downstream of 10,691 annotated genes had increased H3K36me2. Of such genes, 324 were dysregulated in *Kdm8* mutant hearts. This suggests that the histone demethylase activity of Kdm8 influences transcription of a small proportion of its target genes. Nonetheless, such targets represent 70.7% of the 458 genes dysregulated in 2-month-old mutant hearts, pointing to Kdm8 as a key gene expression regulator in the heart at early stages of DCM.

**Extended Data Fig. 5:**
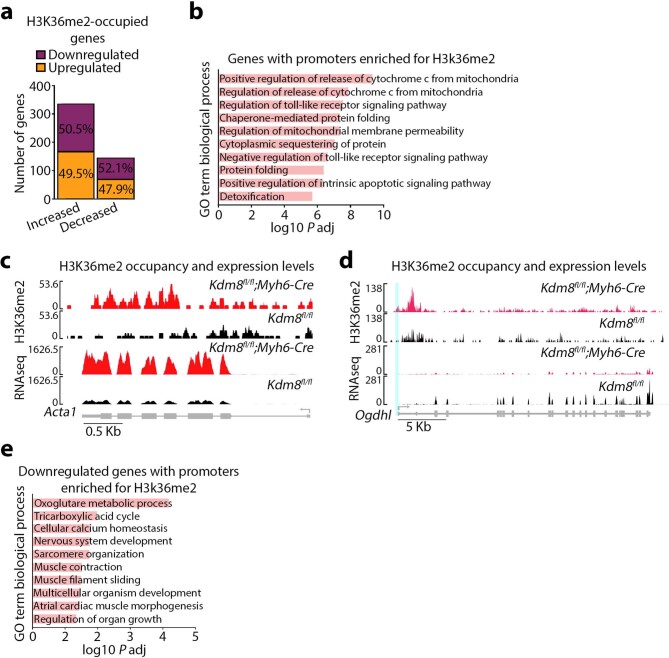
H3K36me2 is enriched at gene bodies of upregulated genes, and promoters of downregulated metabolic genes in *Kdm8* mutant hearts. **a**. Proportion of genes with increased or decreased H3K36me2 that were downregulated or upregulated in 2-month-old *Kdm8*^*fl/fl*^*;Myh6-Cre* mutant hearts. **b**. Gene ontologies (GO) (biological process) enriched amongst genes whose promoters were enriched for H3K36me2 in 2-month-old *Kdm8* mutant hearts. Data was analyzed using DAVID v6.8 with Benjamini correction. **c**. H3K36me2 CUT&Tag and RNAseq tracks of *Acta1* and **d**. *Ogdhl* loci in 2-month-old *Kdm8*^*fl/fl*^ control and *Kdm8*^*fl/fl*^*;Myh6-Cre* mutant hearts. The blue line indicates the promoter region upstream the transcription start site of *Nampt*. Units for CUT&Tag are reads per kilobase per million mapped reads (RPKM) and reads per million mapped reads (RPM) for RNAseq. **e**. GO terms (biological process) enriched amongst genes downregulated in 2-month-old *Kdm8* mutant hearts. Data was analyzed using DAVID v6.8 with Benjamini correction.

H3K36me2 occupied the gene body and intergenic region but peaked at the 5′ regulatory region immediately upstream of the transcription start site, and was depleted at 3′ ends genome-wide (Fig. 2h). Regions with increased or decreased H3K36me2 occupancy were not overtly biased genome-wide toward genes that were downregulated or upregulated in *Kdm8* mutant hearts (Extended Data Fig. 5a). However, H3K36me2 was increased more prominently at promoters (Fig. 2i) of genes enriched for gene ontologies related to mitochondrial function (Extended Data Fig. 5b). H3K36me2 was increased at gene non-coding 5′ regions and, as expected, in the body of genes that were upregulated in *Kdm8* mutant hearts like alpha actin 1 *(Acta1*) (Fig. 2j and Extended Data Fig. 5c). Intriguingly, H3K36me2 enrichment was highest at promoters of downregulated genes (Fig. 2j), including those encoding nicotinamide phosphoribosyltransferase (*Nampt*) and oxoglutarate dehydrogenase l (*Ogdhl*) (Fig. 2k and Extended Data Fig. 5d), which were enriched for functional categories related to metabolic control and muscle contraction (Extended Data Fig. 5e). Accordingly, genes encoding mitochondrial proteins that had increased H3K36me2 at the promoter were expressed at lower levels than those with increased H3K36me2 at the gene body (Fig. 2l). In contrast, all the genes with increased H3K36me2 at the promoter or gene body were expressed at comparable levels (Fig. 2l). Thus, Kdm8-mediated H3K36me2 demethylation preferentially targets promoters of metabolism regulatory genes to maintain their expression in the heart.

### The NAD^+^ synthesis pathway is downregulated as DCM progresses

**Fig. 3:**
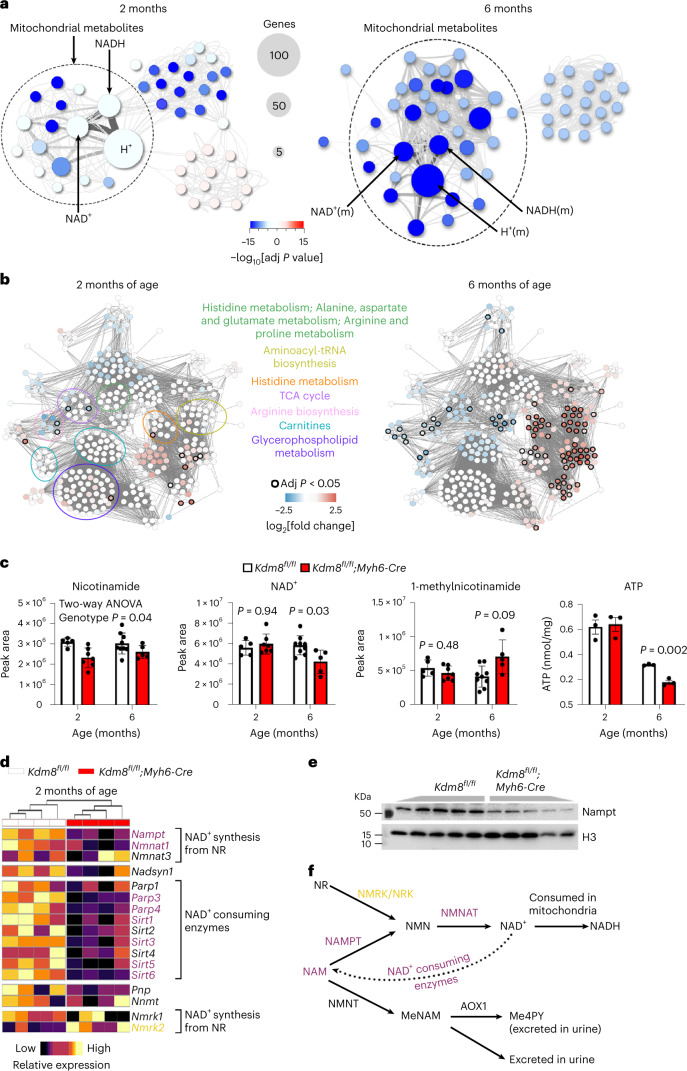
NAD^+^ synthesis metabolome is progressively altered at early stages of DCM. **a**, Reporter metabolite analysis using differentially expressed genes in ventricles of *Kdm8*^*fl/fl*^ control and *Kdm8*^*fl/fl*^*;Myh6-Cre* mutant mice at 2 and 6 months of age. The left panel illustrates all 46 reporter metabolites detected in 2-month-old hearts. The right panel illustrates the top 55 out of 718 reporter metabolites. Upregulated metabolites are in red, and downregulated metabolites are in blue. Data were analyzed by piano using distinct-directional *P* values. *n* = 2 mice per group at 2 months, and 3 mice per group at 6 months. **b**, Metabolomic networks from liquid chromatography–mass spectrometry (LC–MS) analysis of ventricles of control and mutant hearts at 2 and 6 months of age. Significantly altered metabolites are circled in black. Upregulated metabolites are in red, and downregulated metabolites are in blue. Each time point was independently analyzed by two-tailed Student’s *t*-test. *P* values were adjusted for false discovery by the Benjamini–Hochberg method. *n* = 5 controls and 7 mutants at 2 months; *n* = 9 controls and 5 mutants at 6 months. **c**, Quantification of NAM, NAD^+^ and 1-methyl-NAM by LC–MS, and ATP by fluorometric assay. Data are mean ± s.d. Each time point was analyzed by an independent two-tailed Student’s *t*-test, and two-way ANOVA with genotype, time point, and genotype–time point interaction as main effects. *P* values were adjusted for FDR by the Benjamini–Hochberg method. *n* = 5 controls and 7 mutants at 2 months; *n* = 9 controls and 5 mutants at 6 months for LC–MS. *n* = 3 hearts per group for ATP fluorometric assay. **d**, Heatmap of qPCR of genes encoding enzymes in the NAD^+^ pathway in 2-month-old ventricles. *n* = 4 hearts per group for the top 15 genes. *n* = 5 for *Nmrk1* and *Nmrk2*. Significantly downregulated genes are in purple, and significantly upregulated genes are in yellow. **e**, Western blot of Nampt in 6-month-old ventricles. *n* = 5 hearts per group. The experiment was repeated twice with similar results. **f**, NAD^+^ biosynthetic pathways highlighting significantly downregulated and upregulated genes in *Kdm8* mutant hearts. NAM, nicotinamide; NMN, nicotinamide mononucleotide; NR, nicotinamide riboside; MeNAM, methylnicotinamide; NAD^+^, nicotinamide adenine dinucleotide; Me4PY, *N*-methyl-4-pyridone-5-carboxamide. Source data

To uncover the metabolic processes dysregulated at DCM onset and end-stage heart failure, we first performed reporter metabolite analysis^49^ using transcriptomes of hearts of *Kdm8* mutants at 2 and 6 months of age. This analysis links differentially expressed genes with metabolites and metabolic reactions in genomic-scale metabolic networks. A total of 46 metabolites were predicted to be altered at 2 months and 718 metabolites at 6 months, of which 74% and 99%, respectively, were predicted to decrease. Downregulated reporter metabolites were largely localized to the mitochondria, with the largest clusters being downstream of mitochondrial nicotinamide (NAM), including NAD^+^, NADH and H^+^ (Fig. 3a). To experimentally define the altered metabolites, we performed untargeted metabolomics on 2-month-old and 6-month-old control and *Kdm8* mutant hearts. A total of 383 molecular features were detected in control and mutant hearts. The metabolome of mutant hearts was distinct from that of controls already at 2 months, and more markedly at 6 months by principal component analysis (PCA) (Supplementary Fig. 2). Fourteen molecular features were significantly altered at 2 months, and 83 at 6 months (Fig. 3b and Supplementary Table 2). Consistent with the metabolic reporter analysis (Fig. 3a), metabolites of the NAD^+^ pathway were altered in *Kdm8* mutant hearts. NAM was significantly decreased in 2-month-old and 6-month-old mutants vs controls (Fig. 3c). The NAM derivate metabolite NAD^+^ was decreased at 6 months (Fig. 3c). Conversely, 1-methylnicotinamide, which diverts NAM from being recycled back to NAD^+^ (ref. ^50^), tended to increase at 6 months of age (Fig. 3c). ATP levels were decreased at 6 months like NAD^+^ (Fig. 3c). Thus, NAM is decreased at the onset of DCM, and its downstream metabolite NAD^+^ decreases as DCM progresses, consistent with reduced ATP production in *Kdm8* mutant hearts.

**Extended Data Fig. 6:**
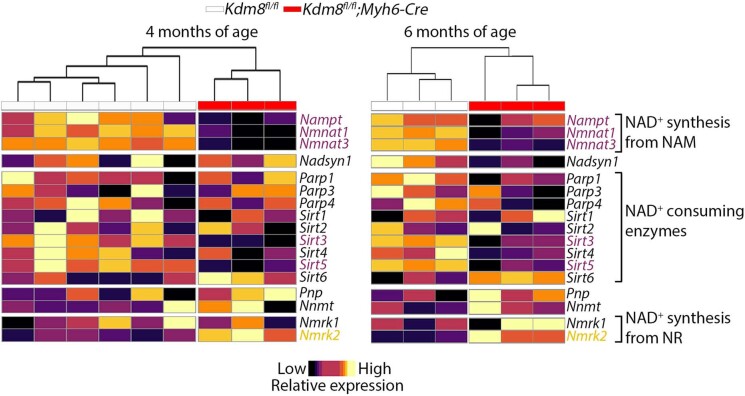
Genes implicated in NAD^+^ synthesis from NAM are repressed during DCM progression. Heatmap of normalized mRNA levels of genes encoding enzymes in the NAD synthesis as per RNA-seq in hearts of *Kdm8*^*fl/fl*^ control and *Kdm8*^*fl/fl*^*;Myh6-Cre* mutant mice at 4 and 6 months of age. Significantly decreased genes are in purple and increased in yellow. Data was analyzed with DESeq2.

To uncover the basis of derangement of the NAD^+^ pathway at the onset and during progression of DCM, we analyzed the expression of genes encoding enzymes in the pathway in 2-month-old, 4-month-old and 6-month-old *Kdm8* mutant hearts (Supplementary Table 1). Notably, the expression of genes encoding proteins regulating the production of NAD^+^ from NAM, such as *Nampt* and *Nmnat1*, which encodes nicotinamide nucleotide adenylyltransferase 1, was decreased at all stages of DCM, and Nampt protein declined at 6 months of age (Fig. 3d,e and Extended Data Fig. 6). Consistent with reduced NAM (Fig. 3c), the expression of genes encoding sirtuins 3 and 5 (*Sirt3* and *Sirt5*), which cleave NAD^+^ back to NAM, was decreased (Fig. 3d and Extended Data Fig. 6). In contrast, expression of the gene encoding nicotine riboside kinase 2 (*Nmrk2*), which converts nicotine riboside to the NAD^+^ precursor nicotine mononucleotide, was upregulated in hearts at all stages of DCM (Fig. 3d and Extended Data Fig. 6). Increased *Nmrk2* could not compensate for persistently downregulated *Nampt* and *Nmnat1*, because NAD^+^ was decreased as DCM progressed (Fig. 3c). Thus, Kdm8 regulates a transcriptional network controlling cardiac NAM metabolism (Fig. 3f), which is altered at the initiation of DCM before ATP and cardiac function decline toward heart failure.

### *Kdm8* coordinates the transcriptional response of the heart to NAD^+^

**Fig. 4:**
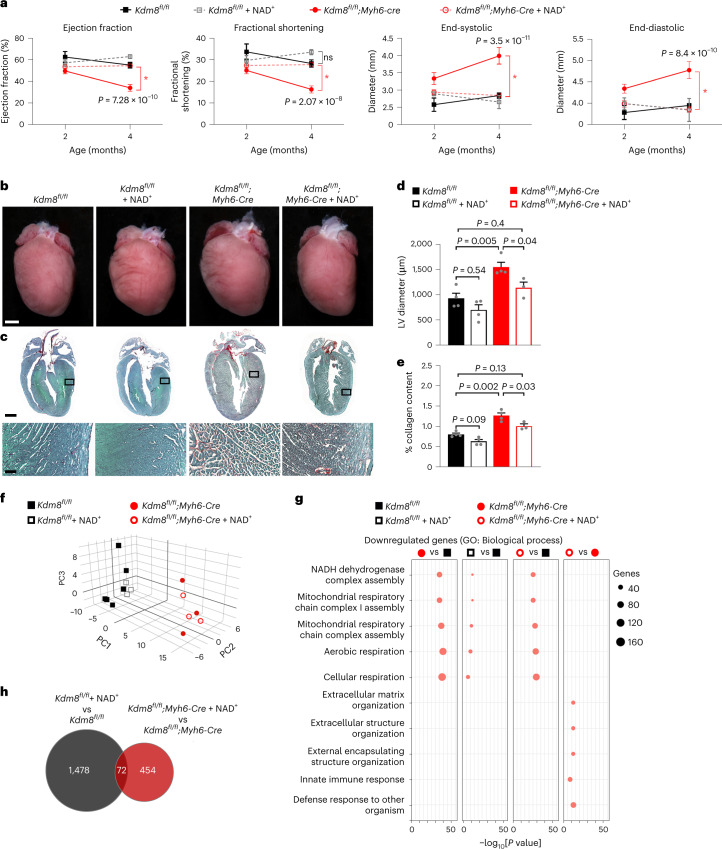
NAD^+^ treatment blunts cardiac deterioration toward heart failure in *Kdm8* mutant mice. **a**, Left ventricle ejection fraction, fractional shortening, and diameter at end-systole and end-diastole obtained from echocardiography on *Kdm8*^*fl/fl*^ control and *Kdm8*^*fl/fl*^*;Myh6-Cre* mutant mice that were untreated or injected with NAD^+^ intraperitoneally for 2 months starting at 2 months of age. Data are mean ± s.e.m. Two-way ANOVA with Tukey’s multiple comparison correction. *n* = 3–9 mice per group (see Source data for details). *Statistically significant. ns = nonsignificant. **b**, Hearts of 4-month-old control and mutant mice that were untreated or treated with NAD^+^. Scale bar, 1 mm. Scale bars = 1 mm and 200 μm. **c**, Micrographs at the top correspond to representative sections of 4-month-old control and mutant hearts that were stained with Sirius Red/Fast Green. Scale bar, 1 mm. Micrographs at the bottom are close-ups of regions in the inset. Scale bar, 100 μm. The experiment was repeated independently on four untreated control, four NAD^+^-treated control, four untreated mutant, and three NAD^+^-treated mutant mice, with similar results. **d**, Diameter of the left ventricle measured from sections of 4-month-old mice. Data are mean ± s.e.m. analyzed by one-way ANOVA with multiple comparison correction by Tukey’s honestly significant difference (HSD) method. *n* is as in **b**. **e**, Percent collagen content in sections of 4-month-old hearts stained with Sirius Red/Fast Green. Data are mean ± s.e.m. analyzed by one-way ANOVA with multiple comparison correction using Tukey’s HSD method. *n* is as in **a**. **f**, Three-dimensional (3D) PCA plot showing variation in the distribution of the transcriptome of untreated and NAD^+^-treated control and mutant hearts. PC, principal component. **g**, GO term enrichment amongst genes downregulated in the heart of NAD^+^-treated control and mutant mice. Four comparisons between control and mutant untreated and NAD^+^-treated mice are indicated. Data were analyzed using g:Profiler. **h**, Venn diagram of genes dysregulated in NAD^+^-treated vs untreated control and *Kdm8* mutant hearts. Source data

NAD^+^ is the hydride acceptor in many oxidation reactions, such as oxidative phosphorylation^50^, and a cofactor of enzymes that regulate gene expression and mitochondrial metabolism^51^. To determine if increasing NAD^+^ availability is sufficient to prevent the onset and/or progression of DCM, we administered NAD^+^ to control and mutant mice daily from 2 to 4 months of age and evaluated cardiac function. To maintain the circadian rhythmicity of NAD^+^, it was injected intraperitoneally every day at the beginning of the night cycle^52^. Consistent with our previous results, cardiac function decreased, and the ventricular diameter and ventricular fibrosis increased in untreated *Kdm8* mutants between 2 and 4 months of age (Fig. 4a–e). Cardiac dimensions and function were not affected in control mice by treatment with NAD^+^ (Fig. 4a). However, cardiac function and ventricular dimensions were not altered (Fig. 4a–c), and interstitial fibrosis was not increased in *Kdm8* mutants treated with NAD^+^ (Fig. 4c,e). Thus, NAD^+^ treatment is sufficient to blunt adverse myocardial remodeling and DCM progression in *Kdm8* mutant mice.

**Extended Data Fig. 7:**
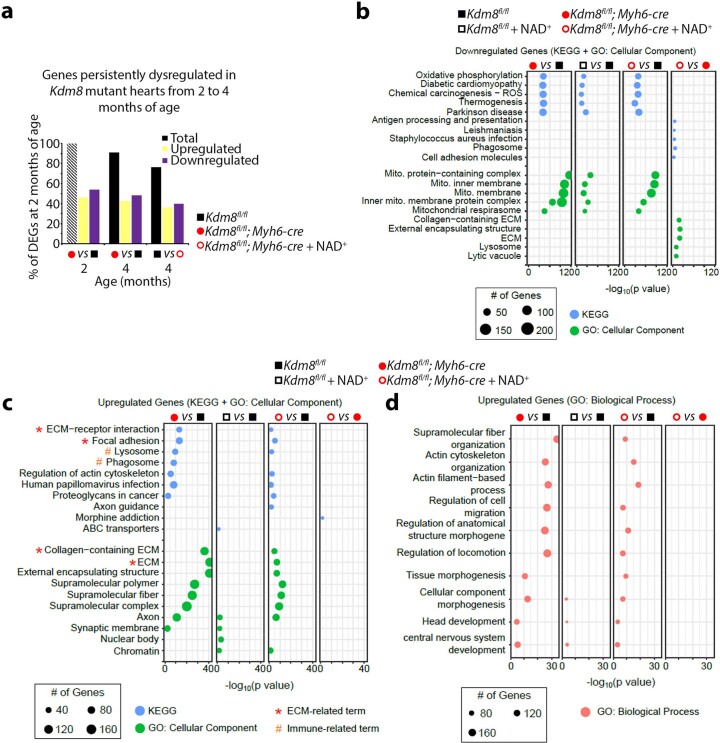
NAD^+^ treatment causes downregulation of genes enriched for functions related to immune responses and extracellular matrix remodelling exclusively in *Kdm8* mutant hearts. **a**, Percentage of differentially expressed genes (DEGs) in 2-month-old *Kdm8*^*fl/fl*^*;Myh6-Cre* mutant hearts at disease onset (total = 458 genes) and that are persistently dysregulated in 4-month old mutant hearts at baseline, and in response to NAD^+^ treatment. Left bars represent the percentage of DEGs that were upregulated or downregulated at 2 months. Middle and right bars represent the percentage of those DEGs that are persistently dysregulated at 4 months. **b**, Gene ontology (GO) terms (cellular component) and KEGG pathways enriched amongst genes downregulated in *Kdm8*^*fl/fl*^ control and *Kdm8*^*fl/fl*^*;Myh6-Cre* mutant mice at baseline and in response to NAD^+^ treatment. **c**, GO term (cellular component) and KEGG pathway (cellular component) enrichment amongst upregulated genes. Data was analyzed using g:Profiler. **d**, GO term (biological process) enrichment amongst upregulated genes. Data was analyzed using g:Profiler

To uncover mediators of cardioprotection by NAD^+^, we analyzed the transcriptome of ventricles of untreated and NAD^+^-treated control and *Kdm8* mutant mice at 4 months of age. The transcriptomes of untreated control and mutant hearts separated on PC1, which explained the largest proportion of variation (Fig. 4f). Notably, the transcriptomes of NAD^+^-treated and untreated mutant hearts clustered together (Fig. 4f) despite normalized cardiac function in NAD^+^-treated mutants (Fig. 4a). Accordingly, over 75% of the 458 genes differentially expressed in 2-month-old mutants at DCM onset (Fig. 2a) were still dysregulated in both NAD^+^-treated and untreated mutant hearts (Extended Data Fig. 7a). This suggests that NAD^+^ does not broadly alter cardiac gene expression, but instead might impact on specific pathways. Before analyzing the effect of NAD^+^, we analyzed the transcriptome of 4-month-old untreated *Kdm8* mutant vs control hearts (Supplementary Table 1) in which the myocardium is deteriorating toward heart failure (Fig. 1g–l). Accordingly, ~10 times more genes (4,303) were dysregulated compared with 2-month-old mutants. Like 2-month-old *Kdm8* mutant hearts, genes downregulated at 4 months were enriched for gene ontologies and pathways linked to oxidative phosphorylation (Fig. 4g and Extended Data Fig. 7b). However, genes predominantly associated with extracellular matrix deposition and immune responses were increased at 4 months of age, but not at 2 months of age (Fig. 2a and Extended Data Fig. 7c,d), supporting ongoing metabolic derangement and adverse myocardial remodeling in progressing DCM.

NAD^+^ had a distinct effect in *Kdm8* mutant compared with control hearts. A total of 1,550 genes were dysregulated in NAD^+^-treated vs untreated control hearts, whereas less than half (527) were dysregulated in NAD^+^-treated vs untreated mutant hearts, with only 72 genes (4%) commonly dysregulated (Fig. 4h). This suggests that Kdm8 coordinates the transcriptional response of the heart to NAD^+^ treatment in the context of heart failure. Genes associated with mitochondrial respiration were predominantly downregulated in both NAD^+^-treated vs untreated control and mutant hearts (Fig. 4g), perhaps reflecting a compensatory adaptation to NAD^+^ excess in treated mice. However, genes predominantly associated with extracellular matrix and immune responses were downregulated exclusively in NAD^+^-treated vs untreated *Kdm8* mutant hearts (Fig. 4g). Thus, NAD^+^-mediated cardioprotection does not involve restoration of transcriptional pathways linked to oxidative metabolism but suppression of pathways linked to secondary extracellular matrix remodeling and inflammatory processes to offset DCM progression.

### *Tbx15* is derepressed in *Kdm8* mutant hearts and blunts NAD^+^-induced cardiomyocyte respiration

**Fig. 5:**
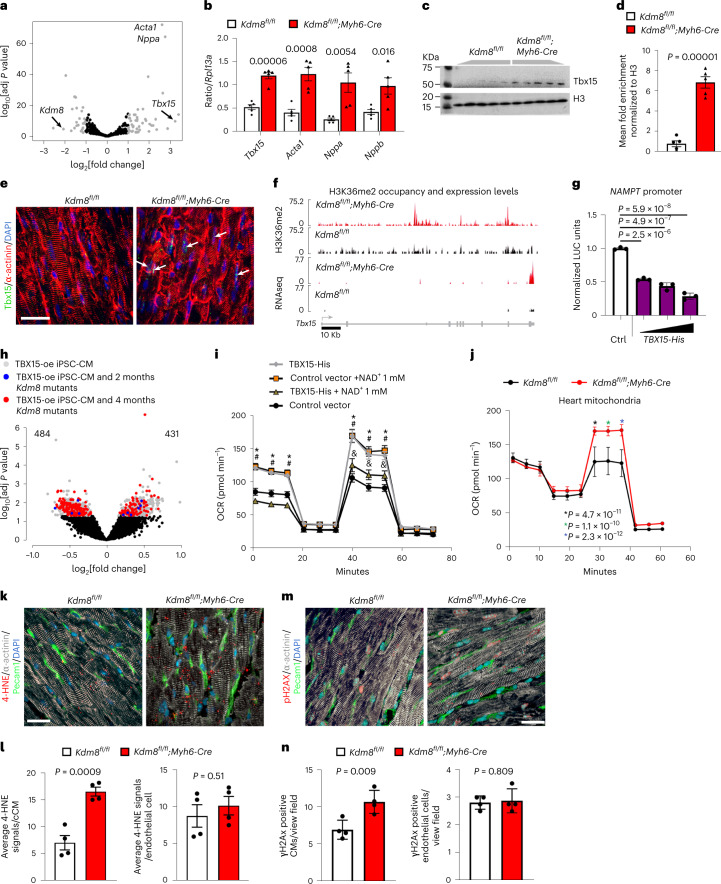
Tbx15 represses a mitochondrial gene network when derepressed in cardiomyocytes. **a**, Differentially expressed genes in 2-month-old *Kdm8*^*fl/fl*^*;Myh6-Cre* mutant vs *Kdm8*^*fl/fl*^ control hearts. Gray dots represent log_2_[fold change] > 1 adjusted *P* < 0.05. *n* = 2 hearts per group. Data were analyzed using DESeq2. **b**, qPCR of *Tbx15*, *Acta1, Nppa* and *Nppb* on ventricles of 2-month-old control and mutant hearts. Data are mean ± s.e.m. Two-tailed Student’s *t*-test. *n* = 5 mice per group. **c**, Western blot of Tbx15 on 6-month-old ventricles. **d**, Protein relative to histone H3. Data are mean ± s.e.m. Two-tailed Student’s *t*-test. *n* = 5 mice per group. **e**, Immunofluorescence of Tbx15 in heart sections. Nuclei were counterstained with DAPI. Scale bar, 20 μm. **f**, H3K36me2 tracks (top) and mRNA fragments (bottom) on *Tbx15* in control and *Kdm8* mutant ventricles. **g**, Luciferase activity of the *Nampt* promoter in HEK293T normalized to pRL-TK, with increasing amounts of a plasmid encoding His-tagged TBX15 (*TBX15-His*). Data are mean ± s.d. One-way ANOVA with Tukey’s multiple comparison correction. *n* = 3 wells per group. **h**, Genes differentially expressed in iPSC-CMs transfected with *TBX15-His*. Gray dots represent dysregulated genes (adjusted *P* < 0.05) in TBX15-overexpressing (oe) iPSC-CMs. Blue dots represent genes dysregulated (adjusted *P* < 0.05) in TBX15-oe iPSC-CMs and 2-month-old *Kdm8* mutant hearts. Red dots represent genes dysregulated (adjusted *P* < 0.05) in TBX15-oe iPSC-CMs and 4-month-old *Kdm8* mutant hearts. Data were analyzed using DESeq2. **i**, Oxygen consumption rate (OCR) of iPSC-CMs transfected with control or *TBX15-His* plasmids and treated with NAD^+^. *, #, and &, *P* < 0.05 (exact *P* values are in Source data). *, comparison between control vector and 1 mM NAD^+^; #, comparison between control vector and TBX15-His; &, comparison between control vector and TBX15-His + 1 mM NAD^+^. Data are mean ± s.e.m. Two-way ANOVA with Tukey’s multiple comparison correction. *n* = 9, 10, 17 and 11 wells for control, 1 mM NAD^+^-treated, TBX15-oe and 1 mM NAD^+^-treated TBX15-oe iPSC-CMs, respectively. **j**, OCR of mitochondria of hearts of 2-month-old control and mutant ventricles. Data represent mean ± s.e. Two-way ANOVA with Sidak correction. *n* = 3 hearts per group. **k**, Immunofluorescence of 4-HNE, α-actinin, Pecam1 and DAPI on sections of 2-month-old control and *Kdm8* mutant hearts. Scale bar, 22 μm. **l**, 4-HNE signals per cardiomyocyte or endothelial cell. Data represent mean ± s.d. Two-tailed Student’s *t*-test. *n* = 4 mice per group. **m**, Immunofluorescence of phosphorylated (Ser139) H2AX (pH2AX), α-actinin, Pecam1 and DAPI on sections of 2-month-old control and *Kdm8* mutant hearts. Scale bar, 22 μm. **n**, Cardiomyocytes, or endothelial cells positive for pH2AX. Data represent mean ± s.d. Two-tailed Student’s *t*-test. *n* = 4 mice per group. Source data

**Extended Data Fig. 8:**
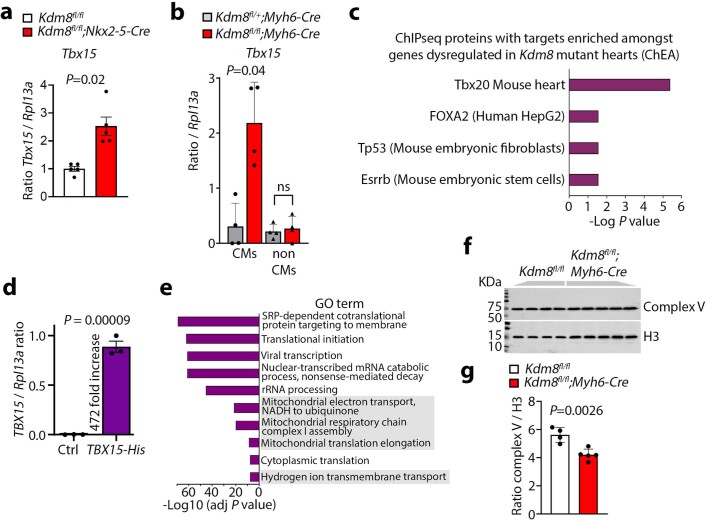
*Tbx15* is derepressed in *Kdm8* mutant cardiomyocytes and represses the expression of metabolic regulators. **a**, qPCR of *Tbx15* on ventricles of *Kdm8*^*fl/fl*^*;Nkx2-5-Cre* mutants at 2 months of age analyzed by two-tailed Student’s t-test. Error bars denote the mean + /- s.e.m. n = 5 hearts per group. **b**, qPCR of *Tbx15* on cardiomyocytes (CMs) and non-CMs isolated from *Kdm8*^*fl/fl*^ control and *Kdm8*^*fl/fl*^*;Myh6-Cre* mutants at 2 months of age. Error bars denote the mean + /- s.d. Data was analyzed by two-way ANOVA. n = 4 mice per group. **c**, Chromatin-bound factors that target genes dysregulated in ventricles of 2-month-old *Kdm8* mutant hearts as per ChIP Enrichment Analysis (ChEA). A complete list of ChEA-identified factors interacting with genes dysregulated in *Kdm8* mutant hearts is shown in Supplementary Table 3. Data was analyzed using ChEA. **d**. qPCR of *TBX15* in HEK293T cells transfected with control or *TBX15-His* plasmids, analyzed by two tailed Student’s t-test. Error bars denote the mean + /- s.e.m. n = 3 wells per group. **e**. Gene ontology (GO) term enrichment amongst genes downregulated in response to *TBX15* overexpression. Terms associated with mitochondrial metabolism are highlighted in grey boxes. Data was analyzed using DAVID v6.8 with Benjamini correction. **f**. Western blot of mitochondrial complex V (ATP5A) in whole ventricles at 2 months of age. **g**, Band intensity of complex V protein normalized to histone H3 (H3) (shown in **f**) and analyzed by two-tailed Student’s t-test. Error bars denote the mean + /- s.d. n = 4 and 5 mice per group. Source data

Epigenetic regulators control cardiac gene expression and homeostasis through key transcription factors^37^. We hypothesized that deregulation of key transcription factors regulated by Kdm8 and controlling NAD^+^ metabolism underlies cardiac deterioration toward heart failure. *Tbx15* was the second most highly upregulated gene in 2-month-old *Kdm8* mutant hearts (Fig. 5a). qPCR confirmed *Tbx15* upregulation comparable to *Acta1*, and natriuretic peptides a and b (*Nppa* and *Nppb*) in *Kdm8*^*fl/fl*^*;Myh6-Cre* (Fig. 5b) and *Kdm8*^*fl/fl*^*;Nkx2-5*-*Cre* mutant hearts (Extended Fig. 8a), and in isolated cardiomyocytes, but not in non-cardiomyocytes of *Kdm8*^*fl/fl*^*;Myh6-Cre* mutants (Extended Data Fig. 8b). Accordingly, western blot and immunofluorescence showed that Tbx15 protein increased by ~7-fold (Fig. 5c,d) and was present exclusively in *Kdm8* mutant cardiomyocyte nuclei (Fig. 5e). Moreover, H3K36me2 occupancy was increased at the gene body of *Tbx15* in *Kdm8* mutant hearts as shown by CUT&Tag (Fig. 5f). ChIP Enrichment Analysis (ChEA)^53^ revealed that genes dysregulated in *Kdm8* mutant hearts were enriched for Tbx20 targets in the mouse heart (Extended Data Fig. 8c and Supplementary Table 3). Consistent with a function for Tbx15 as a transcriptional repressor^38^, a TBX15-His tag fusion protein decreased the activity of the *NAMPT* promoter driving a luciferase reporter in induced pluripotent stem cell-derived cardiomyocytes (iPSC-CMs) in a concentration-dependent manner (Fig. 5g).

Tbx15 represses oxidative metabolism in skeletal muscle and adipocytes^42,44^; however, its function in cardiomyocytes is unknown. We overexpressed a complementary DNA encoding a TBX15-His tag fusion protein in iPSC-CMs by transient transfection (Extended Data Fig. 8d) and analyzed gene expression using RNA-seq. A total of 484 genes were downregulated, and 431 genes were upregulated in TBX15-overexpressing iPSC-CMs (Fig. 5h and Supplementary Table 1). Of the genes downregulated in TBX15-overexpressing iPSC-CMs, 4 genes (~1%) were also downregulated in 2-month-old *Kdm8* mutant hearts, and 163 genes (~34%) were downregulated at 4 months (Fig. 5h). This suggests a predominant function of TBX15 as a transcriptional repressor during DCM progression. Genes downregulated in response to TBX15-His overexpression were enriched for ontologies related to mitochondrial respiration and translation (Extended Data Fig. 8e), similar to *Kdm8* mutant hearts (Fig. 2a). Despite the fact that these results do not directly demonstrate a function of Tbx15 downstream of Kdm8 inactivation, they suggest that *TBX15* overexpression is sufficient to repress regulators of mitochondrial function in cardiomyocytes. Paradoxically, TBX15-His-overexpressing iPSC-CMs had a higher basal and maximal respiration than those transfected with a control vector (Fig. 5i). Such an increase could reflect an adaptation of the stressed mitochondria to glucose utilization that could eventually lead to cardiomyocyte energy exhaustion. Similarly, mitochondria isolated from 2-month-old *Kdm8* mutant hearts also had a higher maximal respiratory capacity than control hearts (Fig. 5j). However, in line with decreased complex V (Extended Data Fig. 8f,g), the increased respiration did not lead to an increased ATP production in 2-month-old mutant hearts, but instead preceded ATP depletion at 6 months (Fig. 3c). Furthermore, cardiomyocytes in 2-month-old *Kdm8* mutants accumulated oxidative damage as shown by increased levels of the lipid peroxidation product 4-hydroxynonenal (4-HNE; Fig. 5k,l) and the marker of oxidative DNA damage phosphorylated H2AX (Fig. 5m,n). Altogether, this suggests that TBX15 represses cardiac metabolic pathways altering the respiratory chain and leading to decreased ATP production when derepressed in cardiomyocytes. Indeed, TBX15-His overexpression blunted the respiration increase induced by NAD^+^ in iPSC-CMs (Fig. 5i). Thus, Kdm8 is required for repression of *Tbx15* to maintain the expression of key mediators of the NAD^+^ pathway to control mitochondrial function and cardiac energy metabolism.

### *TBX15* upregulation and strong downregulation of genes encoding mitochondrial proteins define a group of DCM-affected human hearts

**Fig. 6:**
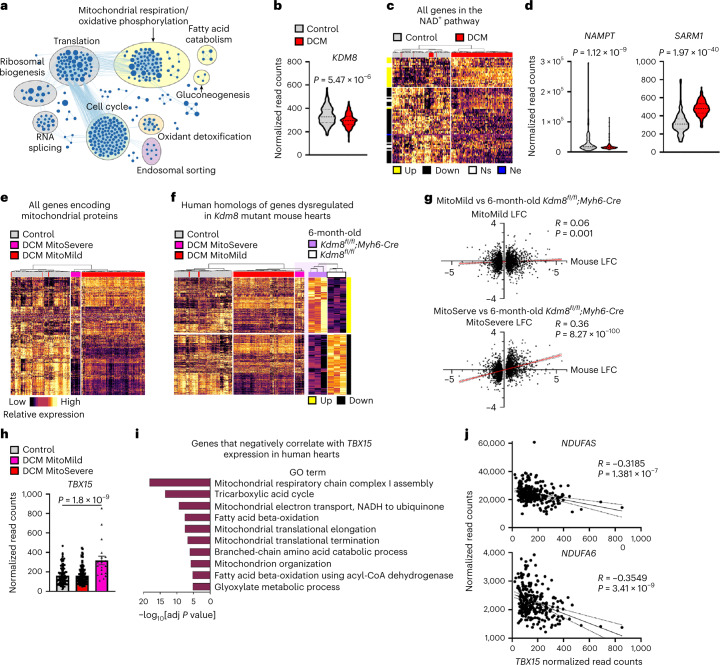
*TBX15* upregulation tracks with stronger downregulation of mitochondrial metabolism regulatory genes in human hearts affected by DCM. **a**, GSEA comparing 149 DCM-affected and 113 control human hearts. Each circle represents a gene set, and blue circles represent downregulated gene sets. All gene sets with Q-value < 0.01 are shown. **b**, Normalized expression of *KDM8* in control and DCM-affected hearts. Thick dotted lines denote the median, and thin dashed lines denote the quartiles. Data were analyzed using DESeq2. **c**, Unsupervised hierarchal clustering of genes in the NAD^+^ pathway. Up, significantly upregulated; Down, significantly downregulated; Ns, not significant; Ne, not expressed. **d**, Normalized expression of *NAMPT* and *SARM1* in control and DCM-affected hearts. Thick dotted lines denote the median, and thin dashed lines denote the quartiles. Data were analyzed using DESeq2. **e**, Unsupervised hierarchal clustering of all genes that encode mitochondrial proteins (obtained from GO term Cellular Component). Three clusters are identified. MitoSevere clusters DCM samples with the lowest expression of mitochondrial genes. **f**, Unsupervised hierarchal clustering of genes dysregulated in 6-month-old mouse hearts (end-stage DCM) shown to the right, and their human homologs shown to the left. The MitoSevere expression profile closely resembles the 6-month-old mutant hearts (light purple background). **g**, Pearson correlation between the log_2_[fold change] (LFC) of dysregulated genes in 6-month-old mutant hearts and of their homologs in the MitoMild (top) or MitoSevere (bottom) samples. Data were analyzed using Pearson correlation two-tailed test. **h**, Normalized expression of *TBX15* in control, MitoMild and MitoSevere hearts. Data are mean ± s.e.m. One-way ANOVA with Tukey’s multiple comparison. *n* = 113 Control, 130 MitoMild, and 19 MitoSevere transcriptomes. **i**, GO term enrichment amongst genes whose expression negatively corelated with *TBX15* expression across all transcriptomes. Correlation was analyzed using Pearson correlation (two-tailed) with Benjamini and Hochberg multiple comparison correction. *n* = 262 transcriptomes. GO enrichment was analyzed using DAVID v6.8 with the Benjamini correction. **j**, Pearson correlation of the expression of *TBX15* with *NDUFA5* and *NDUFA6* across all 262 transcriptomes. The dotted line indicates the 95% confidence interval. Data were analyzed using Pearson correlation (two-tailed) test.

**Extended Data Fig. 9:**
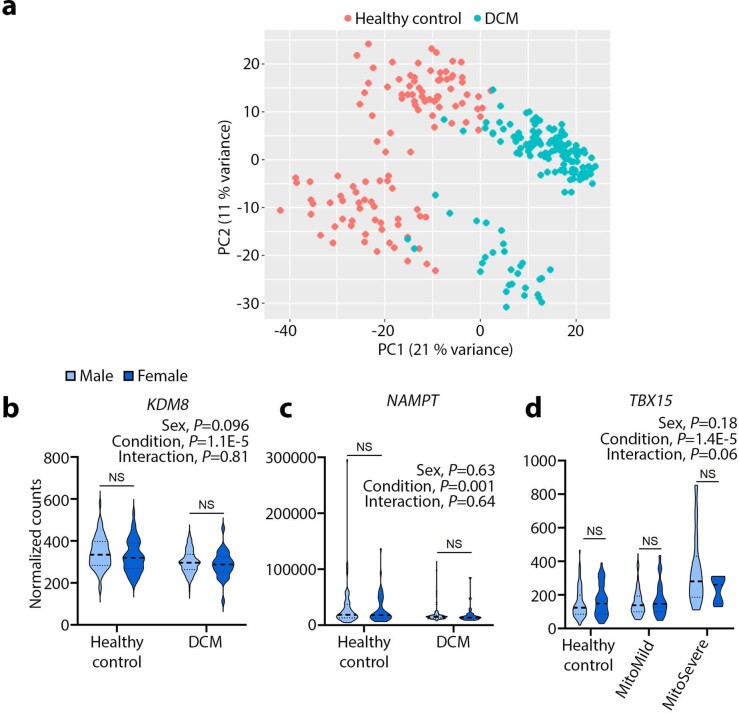
Distinct transcriptional profiles in human hearts affected by dilated cardiomyopathy. **a**, Principal component (PC) analysis plot displaying separation of the transcriptome of healthy human hearts or affected by end-stage dilated cardiomyopathy (DCM). The separation in PC2 is attributed to sex based on the level of transcripts expressed from X and Y chromosomes like *XIST* and *RPS4Y1* respectively. **b**, Violin plots of normalized mRNA levels of *KDM8* and **c**, *NAMPT* in control and DCM-affected human hearts. n = 47 female controls, 66 male controls, 29 female DCM-affected, and 120 male DCM-affected. **d**, Violin plots of normalized mRNA levels of *TBX15* in control, MitoMild and MitoSevere. n = 47 female controls, 66 male controls, 24 female MitoMild, 106 male MitoMild, 5 female MitoSevere, and 14 male MitoSevere. **b**-**d**, Thick dotted lines denote the median. Thin dotted lines denote the quartiles. Data was analyzed by two-way ANOVA and Sidak correction for multiple comparisons.

**Extended Data Fig. 10:**
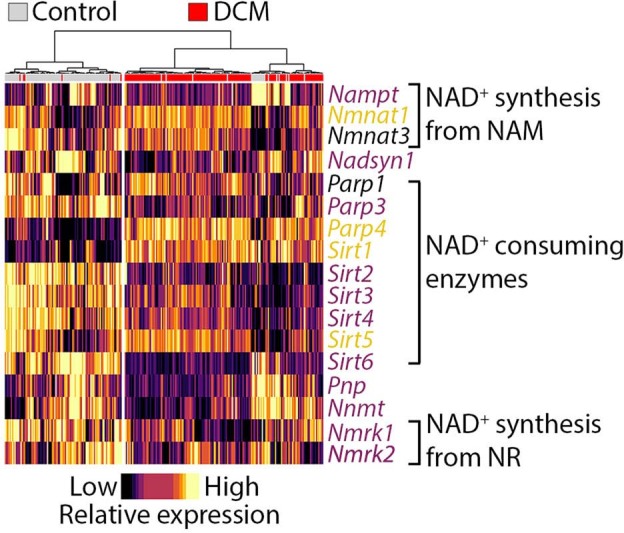
Genes implicated in NAD^+^ synthesis from NAM are repressed in DCM-affected human hearts. Heatmap of normalized mRNA levels of genes encoding enzymes in the NAD synthesis as per RNA-seq in human healthy control and DCM-affected hearts. Significantly decreased genes are in purple and increased in yellow. Data was analyzed with DESeq2.

To assess the regulatory function of a KDM8–TBX15 axis in human DCM, we compared the transcriptome of 149 hearts of people affected by end-stage DCM against 113 healthy controls^54^ (Supplementary Table 1). PCA separated DCM samples from controls (21% variance), indicating distinct transcriptional profiles (Extended Data Fig. 9a). Consistent with sex-biased cardiac gene expression^55^, hearts also separated (11% variance) based on the level of transcripts expressed from the X and Y chromosomes like X-inactive-specific transcript (*XIST*) and ribosomal protein S4 Y-linked 1 (*RPS4Y1*), respectively (Extended Data Fig. 9a). Genes in networks controlling mitochondrial metabolism were predominantly downregulated in DCM-affected hearts (Fig. 6a), coincident with a significant downregulation of *KDM8* (Fig. 6b) in male and female hearts (Extended Data Fig. 9b). Expression analysis focused on genes in the NAD^+^ synthesis pathway revealed that downregulation of this pathway is a hallmark of DCM. Of the genes in the NAD^+^ synthesis pathway, 50% were downregulated, 17% were upregulated and the remainder were either not expressed or not significantly altered in DCM-affected hearts (Fig. 6c). Similar to hearts of *Kdm8* mutant mice (Fig. 2a and Extended Data Fig. 6), genes downregulated in DCM-affected hearts largely encode enzymes required for NAD^+^ production, such as *NAMPT* (Fig. 6d, Extended Data Figs. 9c and 10), whereas upregulated genes predominantly encoded enzymes that divert metabolic precursors away from NAD^+^ synthesis or directly cleave NAD^+^, such as *ACMSD* and *SARM1*, respectively (Fig. 6d and Extended Data Fig. 10). This indicates a transcriptional shift toward reduced NAD^+^ production and availability in hearts at end-stage DCM.

Notably, clustering of all the human samples based on the expression of genes encoding mitochondrial proteins separated DCM-affected hearts from control hearts in two groups hereby referred to as MitoMild and MitoSevere (Fig. 6e). Hearts did not distribute differently between these groups based on sex (MitoMild, 82% males; MitoSevere, 74% males; Fisher’s exact test, *P* = 0.5337). MitoSevere represented ~13% of DCM-affected hearts in which a larger number of genes were more strongly downregulated than in MitoMild (Fig. 6e). Such a broad and strong gene downregulation was recapitulated in 6-month-old *Kdm8* mutant hearts at end-stage DCM. A total of 3,536 genes that were differentially expressed in 6-month-old end-stage *Kdm8* mutant mouse hearts have human homologs. Clustering the human heart transcriptomes based on such homologous genes also separated samples in control, MitoMild and MitoSevere (Fig. 6f). Although the directionality of gene expression change was not always concordant between *Kdm8* mutant hearts and the global human DCM cohort, the MitoSevere cluster almost perfectly recapitulated the expression profile in *Kdm8* mutants (Fig. 6f). Accordingly, the expression of genes dysregulated in 6-month-old *Kdm8* mutants positively correlated with MitoSevere, but not MitoMild (Fig. 6g). Genes in MitoMild or MitoSevere did not correlate with those dysregulated in 2-month-old *Kdm8* mutant hearts (Supplementary Fig. 3). This suggests that MitoSevere might not define a later disease stage and instead perhaps represents a unique form of heart failure underlined by a more profound metabolic derangement. Thus, strong dysregulation of genes encoding mitochondrial proteins defines a subgroup of human hearts at end-stage DCM that is modeled in 6-month-old *Kdm8* mutant hearts.

**Fig. 7:**
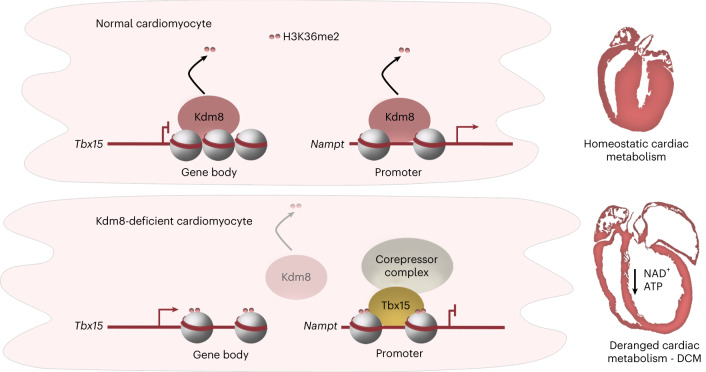
Schematic of the function of Kdm8 in cardiac metabolism control. In normal cardiomyocytes, Kdm8-mediated H3K36me2 demethylation in the *Tbx15* gene body associates with repression. In contrast, H3K36me2 preferentially demethylates promoters of active genes encoding mitochondrial proteins like *Nampt*. In Kdm8-deficient cardiomyocytes, H3K36me2 is enriched in the body of upregulated genes like *Tbx15* and in promoters of genes encoding mitochondrial proteins. Upon gene derepression, Tbx15 with associated corepressor complexes represses genes controlling mitochondrial function, leading to decreased NAD^+^ that precedes ATP depletion and cardiac deterioration toward DCM and heart failure.

A function of TBX15 as a metabolic suppressor downstream of *KDM8* inactivation in human DCM would predict downregulation of genes encoding mitochondrial proteins concordant with higher expression of *TBX15* in affected hearts. Indeed, in both male and female hearts, *TBX15* was expressed at higher levels than controls in MitoSevere, but not in MitoMild (Fig. 6h and Extended Data Fig. 9d). Moreover, 2,128 genes highly enriched for functions related to mitochondrial metabolism, including NADH:ubiquinone oxidoreductase subunit A (*NDUFA5*) and *NDUFA6*, had a weak to strong (−0.2 to −1 correlation coefficient) negative correlation with *TBX15* expression levels (Fig. 6i,j). Conversely, 3,985 positively correlated genes enriched for functions related to inflammatory response, cell adhesion and extracellular matrix organization, amongst others (Supplementary Fig. 4). Thus, repression of genes encoding mitochondrial proteins is a hallmark of end-stage DCM. Furthermore, higher expression of *TBX15* defines a group of DCM-affected hearts with the strongest repression of metabolic gene networks, linking *KDM8* and *TBX15* with heart failure. Altogether, our results suggest that KDM8 epigenetically controls cardiac metabolism repressing *TBX15* to prevent the initiation of cardiac deterioration toward heart failure (Fig. 7).

## Discussion

Histone demethylases modify chromatin in response to the cellular metabolic environment^56^ and are emerging as regulators of heart function. Histone demethylases are differentially expressed in human hearts affected by DCM^57^ and could act as metabolic regulators in heart failure^23^. For example, inactivation of *Smyd1* alters cardiac energetics and DCM^25^. However, the function of histone demethylases and their target histone marks in the heart is still poorly understood.

H3K36me2 is predominantly associated with the body of transcribing genes^58^. Accordingly, we found that H3K36me2 was increased in the body of genes upregulated in *Kdm8* mutant cardiomyocytes like *Tbx15* (Fig. 5f). However, H3K36me2 was most highly enriched in promoters of downregulated genes (Fig. 2j), suggesting a function of this mark in transcriptional repression. Such a repressive function could be mediated by DNA methylation and deacetylation of H3K27. In mouse mesenchymal cells, H3K36me2 mediates recruitment of the DNA methyltransferase DNMT3A to non-coding regions to maintain methylation of intergenic DNA^59^. Moreover, in fission yeast, the H3K36 methyltransferase Set2 cross talks with the histone deacetylase Clr6, which removes H3K27ac at gene promoters, leading to transcriptional repression^31^. Our findings suggest a non-canonical function of H3K26me2 in transcriptional repression preferentially targeting promoters of genes encoding mitochondrial proteins (Fig. 7). Therefore, identifying repressor complexes mediating DNA methylation and H3K27ac deacetylation in Kdm8 target promoters would help uncover pathological mechanisms altering cardiac metabolism. In this regard, the enrichment of TBX targets amongst genes controlling metabolism and that were downregulated in *Kdm8* mutant hearts suggests a potential mechanism of recruitment of repressor complexes. TBX proteins can bind to nucleosomal DNA, suggesting capacity to modify chromatin as pioneer factors^60^. Tbx15, Tbx18 and Tbx20 mediate potent transcriptional repression via corepressor complexes of the Groucho family^38,61^, which interact with histone deacetylases and other chromatin modifiers^62^. For example, Tbx20, which in cardiomyocytes preferentially targets genes dysregulated in *Kdm8* mutant hearts (Extended Data Fig. 8c), recruits NuRD complex components via Groucho-related proteins expressed in the heart^58^. Our finding that Tbx15 acts as a metabolic gene repressor upon Kdm8 inactivation suggests that Kdm8 controls *Tbx15* to maintain metabolic gene expression in the heart. Tbx factors compete for binding sites^38^. Therefore, Tbx15 recruiting corepressors could displace activating complexes associated to other Tbx factors, like Tbx5 (ref. ^38^), when derepressed in the heart. Although control of cardiac metabolism by Kdm8 independent of Tbx15 remains an important possibility, our findings suggest that repression of *Tbx15* in cardiomyocytes is required to maintain the expression of a cardiac metabolic network, and that upon its derepression it downregulates NAD^+^ metabolism to initiate cardiac deterioration toward heart failure (Fig. 7).

Dysregulation of TBX15-controlled gene expression could be relevant in human heart failure. Indeed, *TBX15* was upregulated in human failing hearts with the strongest downregulation of genes encoding mitochondrial proteins (Fig. 6h). Therefore, the extent of dysregulation of genes controlling metabolism in the heart could help explain the vast clinical and potentially etiological heterogeneity of DCM^63^. This knowledge would facilitate more precise classification of multifactorial DCM pathogenesis and the identification of subgroups of patients who may respond more predictably to targeted treatment. Our findings raise the possibility that *TBX15* upregulation could define a form of heart failure with predominant, or even caused by, metabolic derangement and that targeting *TBX15* could maintain cardiac metabolism in heart failure.

*Tbx15* was strongly upregulated in *Kdm8* mutants, and *TBX15* overexpression blunted the activation of cardiomyocyte respiratory capacity in response to NAD^+^ treatment (Fig. 5a,i). Our findings open the possibility that TBX15 or its downstream targets and metabolic pathways could be modulated to prevent cardiac metabolism derangement and heart failure. Indeed, dietary supplementation of nicotine riboside to increase cellular NAD^+^ is being clinically trialed to improve cardiac function in end-stage heart failure (ClinicalTrials.gov identifier NCT04528004). We found that NAD^+^ administration prevented DCM progression when administered before signs of cardiac deterioration first appear in *Kdm8* mutant mice. However, NAD^+^ treatment did not restore expression of metabolic regulators, but rather suppressed pathways associated with secondary adverse myocardial remodeling (Fig. 4g). Therefore, NAD^+^ treatment might not directly impact causative pathways. Instead, targeted boosting of the expression of key metabolic regulators could thwart the initiation of adverse myocardial remodeling. Our finding that increased H3K36me2 was biased toward promoters of downregulated metabolic regulators upon Kdm8 inactivation (Fig. 2l) suggests an important function for Kdm8-mediated demethylation to maintain metabolic gene expression. Therefore, counteracting H3K36me2 methylation of the promoter of metabolic regulators targeted by Kdm8 could be investigated as a strategy to maintain energy homeostasis and prevent the initiation of cardiac deterioration toward heart failure.

Thus, deregulation of a KDM8–TBX15 axis sits high in the hierarchy of events that control the initiation of myocardial remodeling toward DCM and could be targeted to maintain cardiac metabolism and function early in DCM to prevent heart failure.

## Methods

### Mice

All animal procedures were approved by the Animal Care Committee at The Centre for Phenogenomics. The following mouse strains were used: *Kdm8*^*fl/fl*^ (ref. ^35^), *Myh6-Cre* (ref. ^46^) and *Nkx2-5-Cre* (ref. ^48^). *Kdm8*^*fl/fl*^ and *Myh6-Cre* lines were kept in a C57BL6/J background, and *Nkx2-5-Cre* was kept in an ICR background. Mice were housed in standard vented cages in rooms with controlled temperature (20–22 °C) and humidity (40–60%) with 12 h light–dark cycles, and free access to water and food. Mice were fed standard chow (Teklad Global 18% Protein Rodent Diet, ENVIGO, TD.2918X). For sample collection, mice were killed using cervical dislocation. For all experiments, except metabolomics and cardiomyocyte isolations, hearts were injected with 1 M KCl to arrest them in diastole. Hearts were dissected in ice-cold PBS and lightly dried to remove excess PBS prior to weighing. Samples were immediately prepared for downstream analysis thereafter. Only male mice were analyzed in this study. We will analyze female mice soon.

### Echocardiography

Cardiac morphology and function were evaluated using the high-frequency ultrasound imaging system Vevo 2100 (VisualSonics) with a 30 MHz transducer. All mice were scanned under light isoflurane (1.5%) anesthesia within 20–30 min. Mouse body temperature was carefully monitored using a rectal thermometer and maintained between 37.1 °C and 37.9 °C using a heated platform and a heat lamp. Left ventricular dimensions, systolic functions and diastolic functions were measured^64^.

### Cardiomyocyte isolation

Mice were killed using cervical dislocation, and the heart was immediately perfused for cell dissociation^65^. Cardiomyocytes and non-cardiomyocytes were lysed in TRIzol (Invitrogen) for subsequent RNA extraction.

### Histone demethylase assay

Lysates of hearts of 5-month-old *Kdm8*^*fl/fl*^ control and *Kdm8*^*fl/fl*^*;Myh6-Cre* mutant mice were processed using the Histone Demethylase Fluorescent Activity Kit (Invitrogen, EIAHDMF) following the manufacturer’s instructions for a JMJD2A enzyme reaction. We assayed 500 μg of protein in 100 μl reactions in triplicate.

### Metabolomics

#### Sample preparation

Ventricles were dissected in ice-cold PBS to remove blood, weighed and frozen in liquid nitrogen within ~3 min. Ventricles were homogenized in 1.5 ml of extraction buffer (0.1 M formic acid in MeOH, acetonitrile, H_2_O; 40:40:20) per 100 mg of tissue, using a bead homogenizer (TissueLyser II, Qiagen) at 30 kHz for 2 min. A total of 750 μl (equivalent to 50 mg of tissue) of each tissue sample was used for metabolome profiling. An additional 250 μl of extraction buffer were added to bring the volume to 1 ml and prevent freezing. Samples were placed at −20 °C for 30 min and then centrifuged at 20,000*g* at 4 °C for 5 min. The supernatant was transferred to a new tube and stored at −20 °C. The pellet was resuspended in 300 μl of extraction buffer and placed at −20 °C for 30 min, then centrifuged at 20,000*g* at 4 °C for 5 min. The supernatant was transferred and mixed with the supernatant from the previous centrifugation and stored at −20 °C. The pellet was resuspended in 200 μl of extraction buffer, placed at −20 °C for 30 min, and then centrifuged at 20,000*g* at 4 °C for 5 min. The supernatant was transferred and mixed with previous supernatant and stored at −20 °C. We added 41 μl of 6 M ammonium hydroxide per every 2 ml of supernatant to neutralize the pH. Samples were dried in the low-heat setting in a vacuum centrifuge. The pellet was stored at −80 °C.

#### Liquid chromatography–mass spectrometry (LC–MS)

Samples were subjected to chromatographic separation through a Waters ACQUITY BEH HILIC column (3.0 mm × 150 mm, 1.7 μm) connected to a Waters ACQUITY BEH HILIC VanGuard Pre-Column (2.1 mm × 5 mm, 1.7 μm) using a Thermo Scientific UltiMate 3000 ultra-high performance liquid chromatrography (UHPLC) system. The column temperature was maintained at 40 °C, and the autosampler temperature was 10 °C. Injection volume was 10 μl. Eluent A was 20 mM ammonium acetate and 20 mM ammonium hydroxide in water. Eluent B was acetonitrile. The flow rate was 300 μl min^−1^ using the following gradient: 0–1 min, 50% B; 1–2 min, linear gradient to 0% B; 2–10 min, 0% B; 10–10.5 min, linear gradient to 50% B; 10.5–15 min, 50% B. The eluate was directed to a Thermo Scientific Q Exactive Mass Spectrometer with a heated electrospray, HESI II source. Source settings were as follows: Spray Voltage, 3.5 kV; Capillary Temperature, 320 °C; Sheath Gas, 20; Aux Gas, 5; Spare Gas, 2; S-Lens RF level, 55. Full scan spectra was acquired over an *m*/*z* range of 70–1,000 Da at a resolution of 140,000. Both positive and negative polarities were acquired. Data-dependent MS2 spectra were acquired at a resolution of 17,500 using 30 V collision energy.

#### Data processing

Data were analyzed using Thermo Scientific Compound Discoverer v2.1. Metabolites were annotated using the integrated HMDB/KEGG/MetaCyc database, with an error window of 5 ppm.

#### NAD^+^-targeted analysis

A series of serial NAD^+^ dilutions were loaded onto the column. The standard was used to validate retention time and evaluate the response curve. The integrated area for the NAD^+^ peak was based on the retention time of the true standard. Areas were integrated in Thermo Scientific Xcalibur Qual Browser using the ICIS peak algorithm, with a 5 ppm error allowance, and peak smoothing was not enabled.

#### Data analysis

Genotypes were compared by independent two-tailed Student’s *t*-tests for each time point. To account for potential interaction between phenotype and time, two-way analysis of variance (ANOVA) was performed using genotype, time, and genotype–time interaction as main effects. All *P* values were adjusted for FDR using the Benjamini–Hochberg method.

Network analysis was performed using the WGCNA R package. Tightly correlated metabolites were clustered into different modules based on weighted correlation, which raises the correlation using a power threshold. This penalizes weak correlations and maintains strong correlations to reduce spurious correlations for network building. A threshold of 10 was used to achieve a scale-free unsigned network. Singular value decomposition of each module was used to calculate an eigenvalue, which represents the general behavior of the members of each module into a single value.

### Histology

Freshly dissected hearts and lungs in cold PBS were prepared for paraffin embedding^66^. Hearts were sectioned at 7 μm thick sections. Masson’s trichrome (Sigma-Aldrich) and Sirius Red/Fast Green (Chondrex) staining were performed as per the manufacturer’s instructions. Hematoxylin and eosin (H&E) staining was performed as before^66^.

### Immunofluorescence

Dissected tissues were kept in 30% sucrose/PBS at 4 °C overnight. Tissues were embedded in OCT compound and sectioned at 7 μm thickness. Cardiomyocyte cell surface area was measured from heart sections stained with wheat germ agglutinin, Alexa Fluor 594 conjugate (Invitrogen, W11262)^37^. Immunostaining was conducted as before^66^. Antibodies used were COL5A1 (Santa Cruz Biotechnology, sc-20648, 1:200), α-actinin (Sigma-Aldrich, A7811; 1:1,000), TBX15 (Prosci, 30-316; 1:100), CD31 (BD Pharmingen, 553370; 1/100), 4-HNE (Abcam ab46545; 1/100) and phospho-histone H2A.X (Ser139) (203E; Cell Signaling Technology, 9718, 1:100).

### Mitochondrial DNA isolation and quantification

Ventricles were digested in 300 μl of STE buffer containing proteinase K (1 μg μl^−1^) at 50 °C overnight, and then fully homogenized using a handheld homogenizer. Samples were centrifuged at 15,000*g* for 10 min. Supernatant was mixed with 800 μl of isopropanol and centrifuged for 5 min. After removing the supernatant, the DNA pellet was washed with 70% ethyl alcohol and centrifuged for 5 min. Supernatant was removed, and the pellet was dried for 20 min at room temperature before being dissolved in water. DNA was used directly for qPCR using the Advanced qPCR mastermix with SUPERGREEN Lo-ROX (Wisent) on a CFX384 Touch Real-Time PCR Detection System (Bio-Rad). Data were analyzed using CFX Manager software (Bio-Rad). Mitochondrial DNA was normalized against nuclear DNA represented by a region of the β-globin gene locus. Primers are listed in Supplementary Table 4.

### RNA extraction

Whole ventricles were dissected and frozen in TRIzol (Invitrogen) immediately after dissection. Tissue was homogenized at 30 kHz for 2 min using TissueLyser II. Bead-homogenized samples were centrifuged to pellet insoluble tissue. DNA-free RNA was isolated using the Direct-zol RNA MiniPrep Kit (Zymo Research).

### Gene expression analysis by qPCR

cDNA was prepared using the SuperScript III First-Strand Synthesis Kit (Invitrogen) and was used for qPCR. Advanced qPCR mastermix with SUPERGREEN Lo-Rox was used for qPCR on the CFX384 Touch Real-Time PCR Detection System. All samples were run in triplicate. Data were analyzed using CFX Manager. Primers are listed in Supplementary Table 4.

### RNA-seq library preparation

Poly(A) mRNA was isolated from 1 μg of total RNA of adult heart using the NEBNext Poly(A) mRNA Magnetic Isolation Module (New England Biolabs). mRNA was used to prepare RNA-seq libraries using the NEBNext Ultra II RNA Library Prep Kit for Illumina (New England Biolabs). Single-end sequencing (50 bp) was performed on the Illumina HiSeq 2500 or NovaSeq platforms.

### RNA-seq analysis

Sequence reads were trimmed and aligned to the reference mouse genome (mm10 - Ensembl release 104) using Trimmomatic v0.36 and STAR v2.7.9^64^. For analysis of expression in iCell, reads were aligned to the reference human genome (hg38 - Ensembl release 104). For analysis of transcriptomes of human hearts affected by DCM, mapped sequencing reads were from the European Genome-Phenome Archive (EGAS00001002454)^54^. Total counts were quantified using FeatureCounts and were normalized on R v4.0.2 using DESeq2 v1.28.1. Adjusted *P* values < 0.05 were considered significant. Three-dimensional (3D) PCA plots were generated using Plotly in R. Heatmaps were generated with pheatmap, using a Pearson correlation distance measure. Gene Ontology (GO) term and Kyoto Encyclopedia of Genes and Genomes (KEGG) pathway enrichment was performed with DAVID v6.8^67,68^ or gprofiler2 v0.2.1^69^. Volcano plots and heatmaps were created using R. Gene homology between *Homo sapiens* and *Mus musculus* was analyzed using BioMart – Ensembl. Genes related to the NAD^+^ pathway in humans were extracted from the Gene Ontology (GO) project^70^ through manual curation of NAD^+^-related GO terms and aggregating the genes within such terms. Genes encoding mitochondrial proteins in humans were identified from the Cellular Component GO term “mitochondrion” from the GO project.

### Gene set enrichment analysis (GSEA)

GSEA (https://www.broadinstitute.org)^71,72^ was performed^64^ using gene sets “Mouse_GO_AllPathways_no_GO_iea_June_01_2021_symbol.gmt” for mice, or “Human_GO_AllPathways_no_GO_iea_February_01_2022_symbol” for humans, compiled by the Bader Lab at http://download.baderlab.org/EM_Genesets. To identify enriched GO terms and pathways in 2-month-old mouse heart transcriptomes, all expressed genes (~14,000 genes) were ranked by their −log_10_[adjusted *P* value] × (sign of fold change) estimated by DESeq2. To identify enriched GO terms and pathways in human hearts affected with DCM, all expressed genes (~30,000 genes) were ranked, using the same approach. Gene sets smaller than 15 and larger than 500 were excluded from the analysis. For human transcriptome analysis, Cytoscape was used for network visualization^64^.

### CUT&Tag

A total of 100,000 cardiomyocytes isolated from 2-month-old hearts were processed using the CUT&Tag-IT Assay Kit (Active Motif) with the following specifications or modifications: (1) primary antibodies were incubated overnight, (2) the secondary antibody was incubated for 2 h, (3) binding of the pA-Tn5 transposomes with the secondary antibodies was carried out for 2 h and (4) PCR amplification was carried out for 18 cycles. The following antibodies were used: H3K36me2 (Active Motif, 39255; Cell Signaling Technology, 2901; 1 μl of each antibody were combined for each reaction).

### CUT&Tag analysis

Raw sequence reads were trimmed using fastp v0.20.0^73^ and aligned to the reference mouse genome (mm10) using Bowtie 2 v2.1.0^74^. SAM files were converted to BAM files using Sambamba v0.7.1^75^. Total reads were normalized with RPKM using bamCoverage - deepTools v3.5.1^76^. MACS v2.2.7.1^77^ was used for peak calling and differential binding events detection. Homer v4.2^78^ was used for peak annotation and motif enrichment. GO term enrichment amongst annotated peaks was analyzed using the Genomic Regions Enrichment of Annotations Tool (GREAT) v4.0.4^79^. UCSC Table Browser^80^ was used to retrieve genome bed files for mm10. CUT&Tag and RNA-seq tracks are visualized in NGS plotting tool SparK v2.6.2^81^. deepTools v3.5.1^76^ was used to create heatmaps and plot profiles. Genes encoding proteins localized to mitochondria were sourced from MitoCarta3.0^82^.

### ATP quantification assay

Hearts were weighed, and ATP was quantified using the ATP Assay Kit (Abcam, ab83355) following the fluorometric assay. Samples of 2-month-old and 6-month-old hearts were prepared and analyzed in two different batches. Owing to potential batch effects, we performed statistical analysis using an independent two-tailed Student’s *t*-test for each time point. All *P* values were adjusted for FDR using the Benjamini–Hochberg method.

### NAD^+^ treatment in mice

Intraperitoneal injections started at 2 months of age and continued daily until 4 months of age. Each day, NAD^+^ (Sigma-Aldrich, N7004) was solubilized in saline and immediately injected at 50 mg kg^−1^. Injections were performed at the end of the light period (ZT11).

### Reporter metabolite analysis

The R package piano v2.4.0 was used. As the input, we used the 2-month and 6-month mouse heart mRNA fold changes and adjusted *P* values (from DESeq2), and the genome-scale metabolic model of *Mus musculus* v1.3.0^83^. We used 1,000 permutations, and a null distribution for significance assessment of gene sets. We set the smallest and largest gene set sizes allowed in the analysis to 5 and 500, respectively. To define a process or pathway as significant, we used an adj *P* < 0.05 as a cutoff for “distinct direction” of piano.

### Western blot

Ventricles were homogenized in 1 ml of tissue extraction reagent (FNN0071) containing 50 μl ml^−1^ protease inhibitor (P2714) using a bead homogenizer (TissueLyser II) at 30 kHz for 2 min. Samples were then sonicated for 30 s and centrifuged at 13,000*g* for 5 min. Supernatant was collected, and protein concentration was measured using the Pierce BCA Protein Assay Kit (Thermo Scientific, 23227). Lysates were resolved by SDS–PAGE and transferred to a nitrocellulose or PVDF membrane using the Trans-Blot Turbo Transfer System (Bio-Rad). Membranes were blocked in 3% BSA in TBST and then incubated overnight at 4 °C with primary antibodies in TBST containing 1% BSA. Membranes were then washed three times and incubated with secondary antibodies in TBST containing 1% BSA for 1 h. Images and the relative intensities of the immune reactive bands were captured using the Odyssey Fc system (LI-COR Biotechnology). Protein levels of each sample were normalized to H3. Primary antibodies used were anti-H3K36me2 (Active Motif, 39255, 1:1,000), Tbx15 (Prosci, 30-316, 1:500), H3 (Abcam, ab1791, 1:2,000), anti-Nampt (Abcam, ab24149, 1:1,000), Complex I / Ndufb8 (Abcam, ab134367, 1:1,000), Complex IV / Cytochrome C (Santa Cruz Biotechnology, sc13156, 1:1,000), Complex V / ATP5A (Abcam, anti-ATP5A antibody, ab14748, 1:1,000) and Kdm8 (DSHB, PCRP-KDM8-1A2, 1:1,000). All specimens analyzed in each experiment were run in the same gel. Proteins and loading controls were revealed in the same gel for relative protein level quantification.

### Transmission electron microscopy

Left ventricle tissue was fixed in 2% paraformaldehyde and 2.5% glutaraldehyde in 0.1 M sodium cacodylate buffer for 2 h. Samples were then rinsed in buffer, post-fixed in 1% osmium tetroxide in buffer for 90 min, dehydrated in a graded ethanol series (50%, 70%, 90% and 100%, 20 min each) followed by two propylene oxide changes for 30 min, and embedded in Quetol-Spurr resin. Blocks were cured overnight in the oven at 60 °C. Sections 70 nm thick were cut on a Leica EM UC7 ultramicrotome and stained with uranyl acetate and lead citrate. Samples were imaged on a Tecnai T20 transmission electron microscope. Mitochondrial area was calculated as the area occupied by all mitochondria in a micrograph, relative to the total micrograph area. Mitochondria were manually counted per area to assess density.

### iPSC-CM culture, transfection, NAD^+^ treatment and metabolic analysis

Human iPSC-CMs, iCell Cardiomyocytes (Cellular Dynamics International (CDI), C1105), were seeded on XFe96 Cell Culture Microplates (Agilent) and maintained according to the manufacturer’s protocol with the following modifications. (1) Wells were coated with poly-L-lysine (P4707), followed by 0.025% gelatin, 5 μg ml^−1^ laminin and 10 μg ml^−1^ fibronectin in PBS at 37 °C for 2 h. (2) We seeded 30,000 cells in each well. Cells were transfected using TransIT-LT1 Transfection Reagent (Mirus) after 1 day of culture at a 1:2 ratio of DNA:TransIT-LT1 Reagent. Cells were transfected with TBX15 OHu33746C pcDNA3.1(+)-N-6His (GenScript, OHu33746C), or a GFP-expressing vector as control. Media were changed 2 days after transfection.

For NAD^+^ treatment, culture media with NAD^+^ 1 mM were added to the cells on day 4. NAD^+^-containing media were refreshed on day 6. Metabolism was assessed using the Seahorse XFe96 Analyzer (Agilent) on day 7, following protocols provided by the iCell manufacturer.

### Dual luciferase assay

HEK293 cells were cultured in DMEM (Invitrogen) with 10% FBS (Sigma) on gelatin-coated 96-well plates. Cells were transfected with three plasmids: (1) GFP-OE, pRL-TK and NAMPT-luciferase (Gen-Mm-Nampt-Promoter-1594266219, Bio Basic); or (2) TBX15-OE, pRL-TK and NAMPT-luciferase. Cells were transfected using TransIT-LT1 Transfection Reagent, using a 4:6 ratio of DNA:TransIT-LT1 Reagent. A total of 350 ng of plasmid DNA was consistently transfected. The ratios of TBX15-OE:pRL-TK:NAMPT-luciferase plasmids were adjusted between 0.5:1:1, 1:1:1 or 2:1:1. Luminescence was measured 20 h after transfection using the Dual-Luciferase Reporter Assay System (Promega, E1910) on the Varioskan LUX Plate Reader. All luciferase measurements were normalized to *Renilla reniformis*.

Gen-Mm-Nampt-Promoter-1594266219 (Bio Basic) contains a DNA fragment corresponding to nucleotides −2233 to +63 relative to the transcription start site of the mouse *Nampt* locus cloned upstream luciferase in pGL4.19 Luciferase Reporter Vector (Promega).

### Isolation of heart mitochondria and mitochondrial respiration assay

Mitochondria from ventricles were isolated by differential centrifugation^84^. The hearts were dissected, minced into small pieces and rinsed free of blood in 5 ml of ice-cold PBS with 10 mM EDTA. The minced hearts were resuspended in 5 ml of PBS with 10 mM EDTA and 0.05% trypsin for 30 min on ice, and then centrifuged at 200*g* for 5 min. The pellet was resuspended in mitochondria isolation buffer (50 mM 1 M Tris-HCl, 50 mM 1 M KCl, 10 mM EGTA, 90 mM sucrose and 0.2% BSA, pH 7.4) and homogenized stroking 15 times in a Dounce homogenizer. Following centrifugation at different speeds at 4 °C, the pellet containing mitochondria was resuspended in mitochondria isolation buffer. The total mitochondrial protein was determined using the Pierce BCA Protein Assay Kit. Finally, mitochondria adjusted to 1.0 μg per well were subjected to a coupling assay in a Seahorse XFe/XF96 Analyzer (Aglient) with 250 μM ADP, 2.5 μg ml^−1^ oligomycin, 2.2 μM ml^−1^ FCCP and 2 μM ml^−1^ rotenone^85^. Data were analyzed by two-way ANOVA followed by Sidak correction.

### Statistics and reproducibility

Data are presented as mean ± s.e.m. unless otherwise indicated. Two-group comparisons were conducted by two-tailed Student’s *t*-test. For multiple-group comparisons and for comparisons of two or more groups over time, one-way and two-way ANOVA were used, respectively. Multiple hypothesis testing correction was performed using Tukey’s test for one-way ANOVA and Sidak correction for two-way ANOVA, unless otherwise indicated. *P* < 0.05 was considered significant. Survival curve statistics were calculated using log-rank (Mantel–Cox) test. Statistical analysis was conducted using either GraphPad Prism 8 or R.

qPCR, immunofluorescence, metabolic activity (Seahorse) assays and western blots were repeated at least twice. The results presented are representative of all biological replicates and repeat experiments.

### Reporting summary

Further information on research design is available in the Nature Portfolio Reporting Summary linked to this article.

### Supplementary information

Supplementary Information Supplementary Figs. 1–4.

Reporting Summary

Supplementary Tables 1–4.

### Source data

Source Data Fig. 1 Statistical source data.

Source Data Fig. 2 Unprocessed western blots.

Source Data Fig. 3 Unprocessed western blots.

Source Data Fig. 4 Statistical source data.

Source Data Fig. 5 Unprocessed western blots.

Source Data Fig. 5 Statistical source data.

Source Data Extended Data Fig. 1 Unprocessed western blots.

Source Data Extended Data Fig. 3 Statistical source data.

Source Data Extended Data Fig. 8 Unprocessed western blots.

## Data Availability

The authors declare that the data supporting the findings of this study are available within the paper and its Supplementary Information. The RNA sequencing data and CUT&Tag data sets reported in this study have been deposited in the Gene Expression Omnibus under the accession numbers GSE215351 and GSE215793.

## Code availability

Code for R packages DESeq2, piano, gprofiler2, pheatmap and ggplot2 is publicly available at https://cran.r-project.org. Code for fastp, Bowtie 2, Sambamba, MACS, deepTools, Homer and SparK is publicly available at https://github.com and http://homer.ucsd.edu/homer.
