# Supplementary material for: KDM8 epigenetically controls cardiac metabolism to prevent initiation of dilated cardiomyopathy
Source: Nat Cardiovasc Res. 2023 Feb 13;2(2):174–91. doi: 10.1038/s44161-023-00214-0 (PMC11041705; doi:10.1038/s44161-023-00214-0)
Supplement: Supplementary file 1 — Supplementary Figs. 1–4. [file 44161_2023_214_MOESM1_ESM.pdf]

nature cardiovascular research

Article https://doi.org/10.1038/s44161-023-00214-0

KDM8 epigenetically controls cardiac metabolism to prevent initiation of dilated cardiomyopathy

In the format provided by the authors and unedited

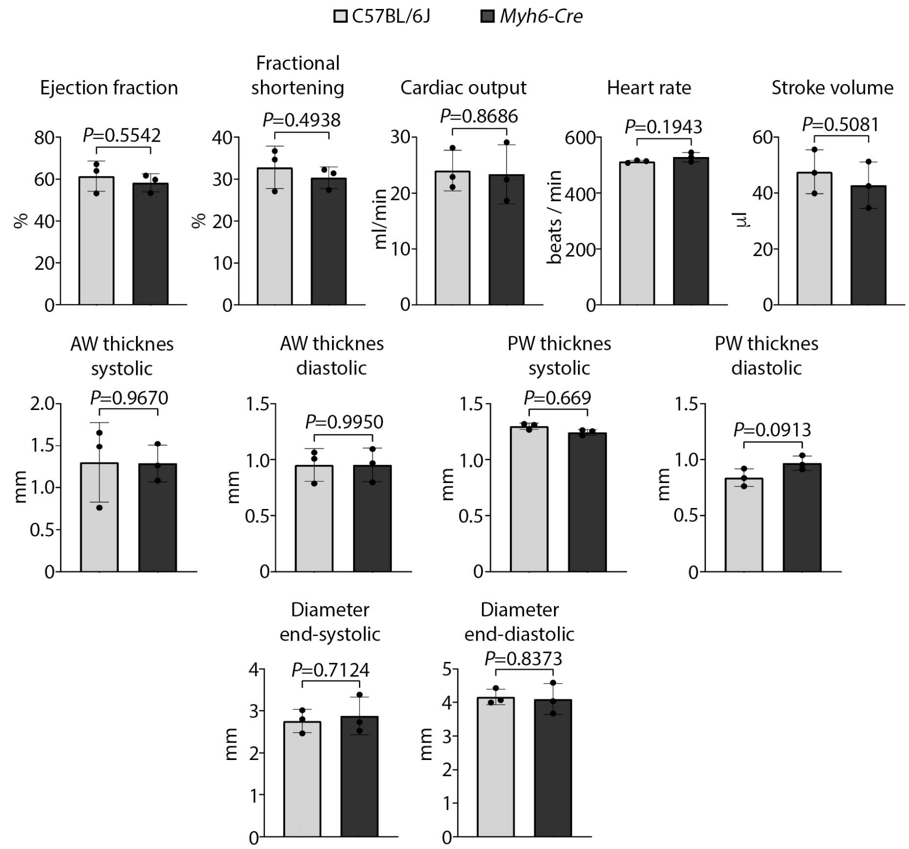

Supplementary Fig. 1. Myh6-Cre does not alter cardiac function in mice at 6 months of age. Echocardiogram analysis of left ventricle ejection fraction, fractional shortening, cardiac output, heart rate, stroke volume, anterior wall (AW) and posterior wall (PW) thickness at end-systole and end-diastole, and diameter at end-systole and end-diastole in control C57BL/6/J and Myh6-Cre transgenics at 6 months of age. Data represent the mean +/- s.d. of three mice per group and was analyzed by unpaired two-tailed Student's t-test.

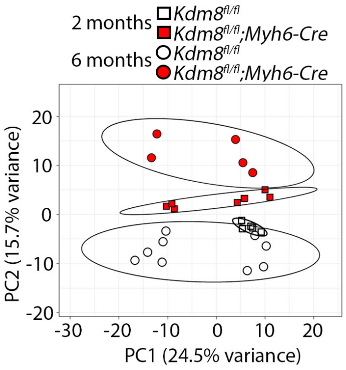

Supplementary Fig. 2. The cardiac metabolome is progressively altered beginning at the onset of DCM. Principal component (PC) analysis plot displaying separation of the cardiac metabolome of *Kdm8*$^{fl/fl}$ control and *Kdm8*$^{fl/fl}$*;Myh6-Cre* mutant mice at 2 and 6 months of age. n = 5 controls and 7 mutants at 2 months; n = 9 control and 5 mutants at 6 months. Circles represent 90% confidence interval.

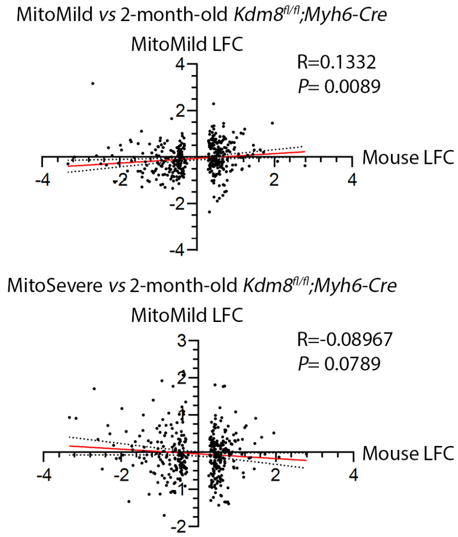

Supplementary Fig. 3. Gene dysregulation at DCM onset in mouse hearts weakly correlates with gene dysregulation in human hearts in the MitoMild group. Pearson correlation between the Log_2 Fold Change (LFC) of dysregulated genes in 2-month-old *Kdm8* mutant hearts, and the LFC of their homologs in MitoMild (top) or MitoSevere hearts (bottom). Data was analyzed using Pearson correlation two-tailed test. Correlation was analyzed using Pearson correlation (two-tailed) with Benjamini and Hochberg multiple comparison correction. n = 262 transcriptomes. GO enrichment was analyzed using DAVID v6.8 with Benjamini correction.

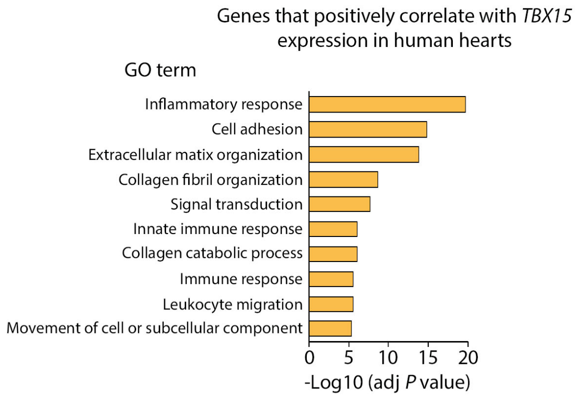

Supplementary Fig. 4. *TBX15* expression positively correlates with the expression of regulators of inflammation and extracellular matrix in human hearts. Gene ontology (GO) terms enriched amongst genes whose mRNA levels positively correlate with *TBX15* expression in human hearts affected by DCM as per RNAseq analysis.
