# Supplementary material for: KDM8 epigenetically controls cardiac metabolism to prevent initiation of dilated cardiomyopathy
Source: Nat Cardiovasc Res. 2023 Feb 13;2(2):174–91. doi: 10.1038/s44161-023-00214-0 (PMC11041705; doi:10.1038/s44161-023-00214-0)
Supplement: Supplementary file 2 — Reporting Summary [file 44161_2023_214_MOESM2_ESM.pdf]

nature portfolio

Corresponding author(s): Paul Delgado Olguin

Last updated by author(s): Dec 30, 2022

Reporting Summary

Nature Portfolio wishes to improve the reproducibility of the work that we publish. This form provides structure for consistency and transparency in reporting. For further information on Nature Portfolio policies, see our Editorial Policies and the Editorial Policy Checklist.

Statistics

For all statistical analyses, confirm that the following items are present in the figure legend, table legend, main text, or Methods section.

n/a	Confirmed	
☐	☒	The exact sample size (n) for each experimental group/condition, given as a discrete number and unit of measurement
☐	☒	A statement on whether measurements were taken from distinct samples or whether the same sample was measured repeatedly
☐	☒	The statistical test(s) used AND whether they are one- or two-sided<br>*Only common tests should be described solely by name; describe more complex techniques in the Methods section.*
☐	☒	A description of all covariates tested
☐	☒	A description of any assumptions or corrections, such as tests of normality and adjustment for multiple comparisons
☐	☒	A full description of the statistical parameters including central tendency (e.g. means) or other basic estimates (e.g. regression coefficient) AND variation (e.g. standard deviation) or associated estimates of uncertainty (e.g. confidence intervals)
☐	☒	For null hypothesis testing, the test statistic (e.g. F, t, r) with confidence intervals, effect sizes, degrees of freedom and P value noted<br>*Give P values as exact values whenever suitable.*
☒	☐	For Bayesian analysis, information on the choice of priors and Markov chain Monte Carlo settings
☐	☒	For hierarchical and complex designs, identification of the appropriate level for tests and full reporting of outcomes
☐	☒	Estimates of effect sizes (e.g. Cohen's d, Pearson's r), indicating how they were calculated

Our web collection on statistics for biologists contains articles on many of the points above.

Software and code

Policy information about availability of computer code

Data collection	No code was used to collect data
Data analysis	R v4.0.2 was used to analyze data. Deseq2 v1.28.1 was used to analyze RNAseq data. piano v2.4.0 was used to perform reporter matabolite analysis. gprofiler2 v.0.2.1 was used to perform enrichment analysis on RNAseq data. The aforementioned R packages are publicly available (https://cran.r-project.org/).

For manuscripts utilizing custom algorithms or software that are central to the research but not yet described in published literature, software must be made available to editors and reviewers. We strongly encourage code deposition in a community repository (e.g. GitHub). See the Nature Portfolio guidelines for submitting code & software for further information.

Data

Policy information about availability of data

All manuscripts must include a data availability statement. This statement should provide the following information, where applicable:

- Accession codes, unique identifiers, or web links for publicly available datasets
- A description of any restrictions on data availability
- For clinical datasets or third party data, please ensure that the statement adheres to our policy

The RNA sequencing data and CUT&Tag data sets reported in this study have been deposited in the Gene Expression Omnibus under the accession numbers GSE215351 and GSE215793.

Human research participants

Policy information about studies involving human research participants and Sex and Gender in Research.

Reporting on sex and gender	Our RNAseq analysis was performed on previously published data of male and female human hearts. In our study, sex was identified based on the level of transcripts expressed from the X and Y chromosomes like XIST and RPS4Y1, respectively. We report the sex-related differences found in our study.<br>We used de-identified transcriptomics data in our analysis.
Population characteristics	*Describe the covariate-relevant population characteristics of the human research participants (e.g. age, genotypic information, past and current diagnosis and treatment categories). If you filled out the behavioural & social sciences study design questions and have nothing to add here, write "See above."*
Recruitment	*Describe how participants were recruited. Outline any potential self-selection bias or other biases that may be present and how these are likely to impact results.*
Ethics oversight	*Identify the organization(s) that approved the study protocol.*

Note that full information on the approval of the study protocol must also be provided in the manuscript.

Field-specific reporting

Please select the one below that is the best fit for your research. If you are not sure, read the appropriate sections before making your selection.

☒ Life sciences ☐ Behavioural & social sciences ☐ Ecological, evolutionary & environmental sciences

For a reference copy of the document with all sections, see nature.com/documents/nr-reporting-summary-flat.pdf

Life sciences study design

All studies must disclose on these points even when the disclosure is negative.

Sample size	Sample size was determined based the expected effect size, expected variation, sample availability and resource constraints. Due to the use genetically homogeneous mouse samples, the biological variation between replicates was minimized, and largely influenced by experimental technique, and instrument limitations. As such, a small sample size of 3 - 5 mice per group was used for most experiments.
Data exclusions	No data was excluded from the analysis.
Replication	Experiements were performed at least three times. Several experiments were replicated (including westerns, echocardiograms, and qPCRs) by more than one individual to ensure reproducibility of results. All qPCR samples were run in 2 to 3 technical replicates. Each finding was supported by multiple experiments to ensure reproducibility of the same result, by leveraging different methods (e.g., gene expression data was supported by RNAseq and qPCR).
Randomization	Samples were taken from different litters and randomly allocated based on the experiment.
Blinding	Mice were identified by a unique alpha-numeric code with no genotype identifier to ensure blinding. After data collection was complete, samples were identified and allocated to the apporporiate gentyping group to enable statistical analysis, as required.

Reporting for specific materials, systems and methods

We require information from authors about some types of materials, experimental systems and methods used in many studies. Here, indicate whether each material, system or method listed is relevant to your study. If you are not sure if a list item applies to your research, read the appropriate section before selecting a response.

Materials & experimental systems

n/a	Involved in the study	
☐	☒	Antibodies
☐	☒	Eukaryotic cell lines
☒	☐	Palaeontology and archaeology
☐	☒	Animals and other organisms
☒	☐	Clinical data
☒	☐	Dual use research of concern

Methods

n/a	Involved in the study	
☒	☐	ChIP-seq
☒	☐	Flow cytometry
☒	☐	MRI-based neuroimaging

Antibodies

Antibodies used	anti-H3K36me2 (Active Motif, 39255), Tbx15 (ProSci, 30-316), H3 (abcam, ab1791), anti-Nampt (abcam, ab24149), Complex I / Ndufb8 (abcam, ab134367), Complex IV / Cytochrome C (Santa Cruz, sc13156), Complex V / ATP5A (abcam, Anti-ATP5A antibody, ab14748), Kdm8 (DSHB, PCRP-KDM8-1A2), were COL5A1 (Santa Cruz Biotechnology, sc-20648, 1:200), alpha-actinin (Sigma-Aldrich, A7811; 1:1000), TBX15 (ProSci, 30-316; 1:100), CD31 (BD Pharmingen, 553370; 1/100), 4-HNE (Abcam ab46545; 1/100), and Phospho-Histone H2A.X (Ser139) (203E; Cell Signaling Technology, 9718, 1:100).
Validation	All antibodies used were validated on the manufacturer's website and have been extensively referenced. Our own experiments validated many of these antibodies, which revealed changes in protein abundance in agreement with RNAseq and qPCR.

Eukaryotic cell lines

Policy information about cell lines and Sex and Gender in Research

Cell line source(s)	HEK293 (Acquired from ATCC)<br>Human induced pluripotent stem cell-derived cardiomyocytes, iCell Cardiomyocytes (Cellular Dynamics International, Inc., (CDI), Madison, WI, USA; C1105)
Authentication	HEK293 were acquired from a commercial vendor.<br>iCells were authenticated by observing rhythmic contraction of the cells in culture.
Mycoplasma contamination	Cell lines were not tested for mycoplasma contamination.
Commonly misidentified lines (See ICLAC register)	*Name any commonly misidentified cell lines used in the study and provide a rationale for their use.*

Animals and other research organisms

Policy information about studies involving animals; ARRIVE guidelines recommended for reporting animal research, and Sex and Gender in Research

Laboratory animals	All animal procedures were approved by the Animal Care Committee at The Centre for Phenogenomics. The following mouse strains were used: Kdm8fl/fl, Myh6-cre and Nkx2-5-cre. Kdm8fl/fl and Myh6-cre lines were kept in a C57BL6/J background, and Nkx2-5-cre in ICR background. Mice were housed in standard vented cages in rooms with controlled temperature (20–22°C) and humidity (40-60%) with 12-hour light-dark cycles, and free access to water and food. Mice were fed standard chow (Tekland Global 18% Protein Rodent Diet, ENVIGO, TD.2918X).
Wild animals	The study did not involve any wild animals.
Reporting on sex	All analysis was conducted on male mice due to a) resource constraints and b) established research data indicating that females have some degree protection from heart disease compared to males. In the Methods sections we specify that only males mice were analyzed in this study.
Field-collected samples	The study did not involve field collections.
Ethics oversight	All animal procedures were approved by the Animal Care Committee at The Centre for Phenogenomics.

Note that full information on the approval of the study protocol must also be provided in the manuscript.
