# Supplementary figures and images for: KDM8 epigenetically controls cardiac metabolism to prevent initiation of dilated cardiomyopathy
Source: Nat Cardiovasc Res. 2023 Feb 13;2(2):174–91. doi: 10.1038/s44161-023-00214-0 (PMC11041705; doi:10.1038/s44161-023-00214-0)

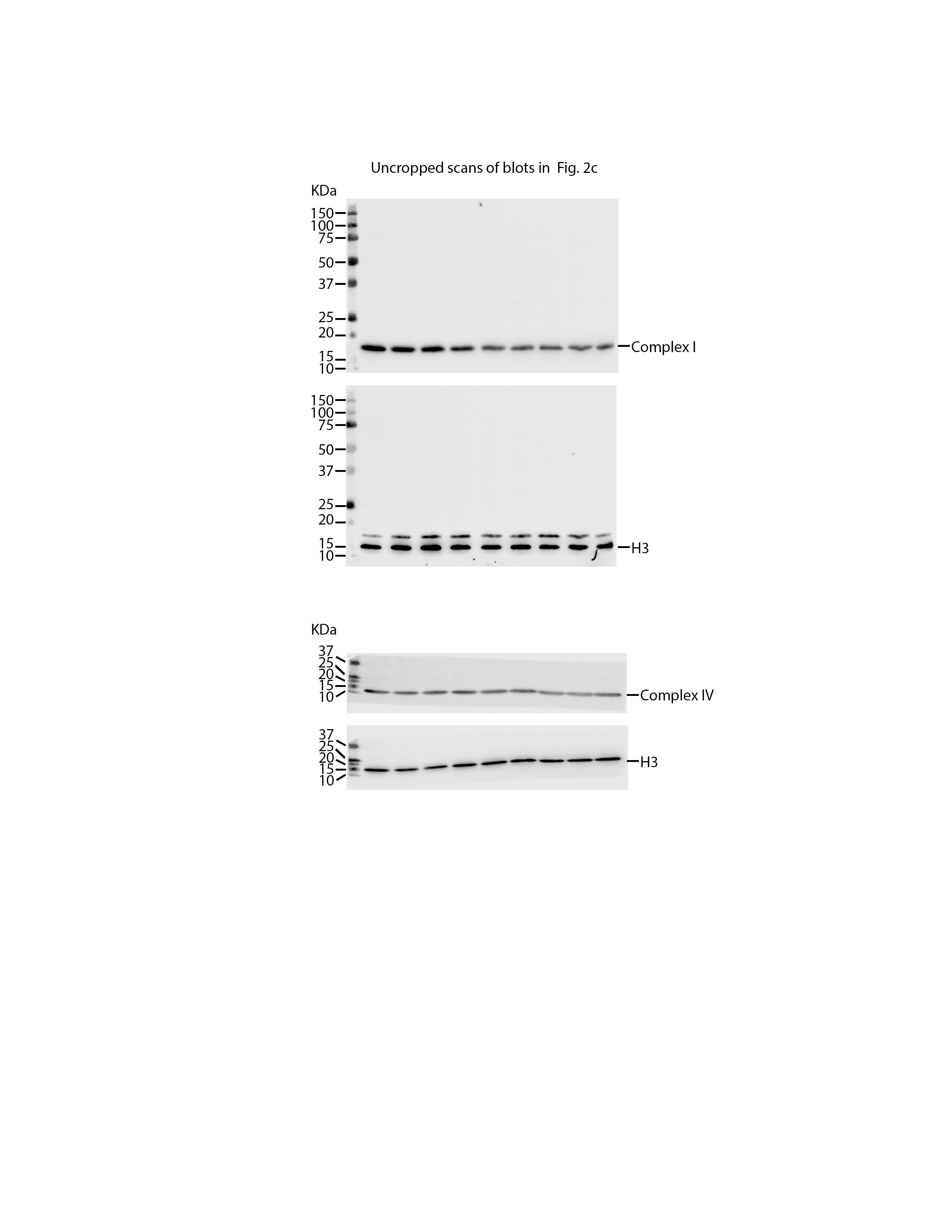

Supplement: Supplementary file 5 — Unprocessed western blots. [file 44161_2023_214_MOESM5_ESM.jpg]

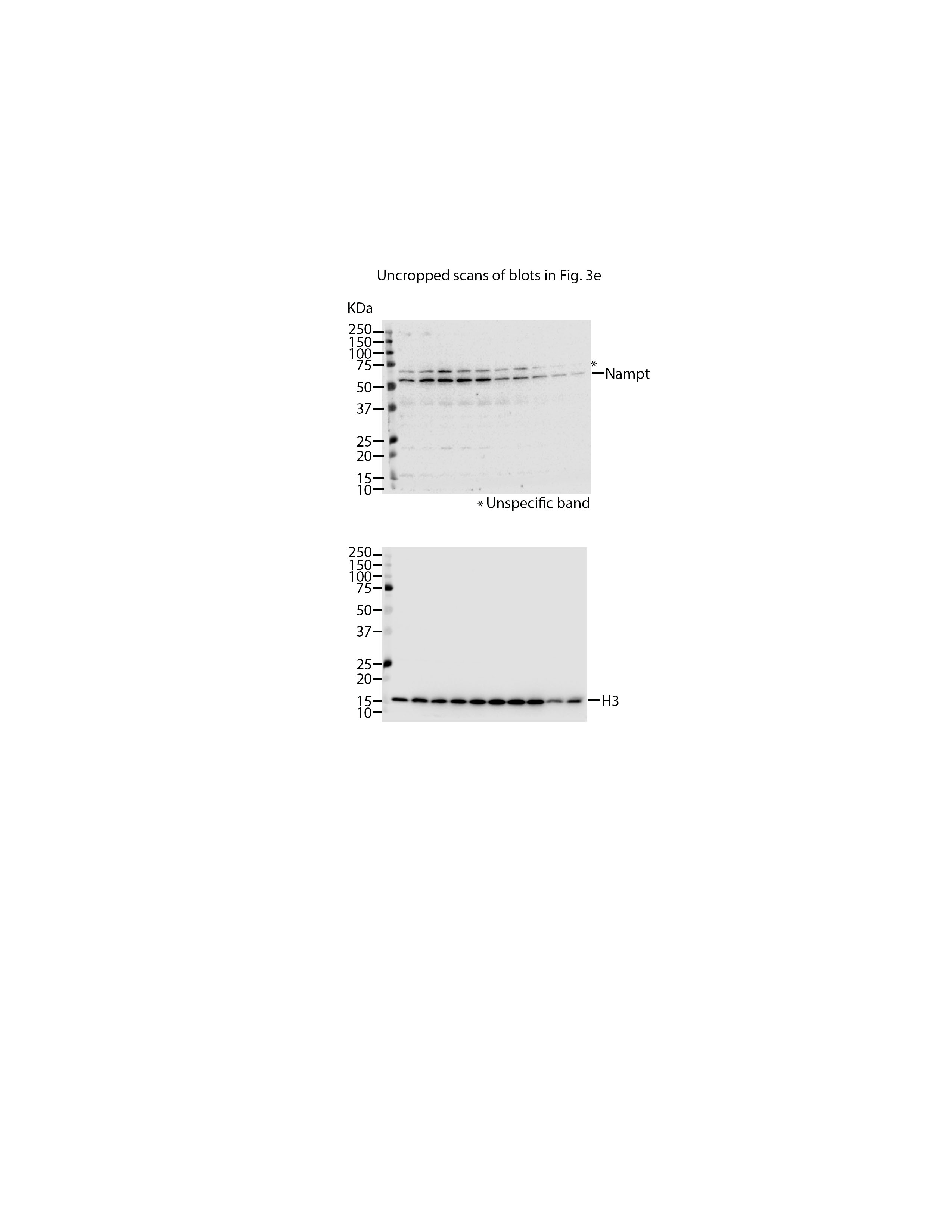

Supplement: Supplementary file 6 — Unprocessed western blots. [file 44161_2023_214_MOESM6_ESM.jpg]

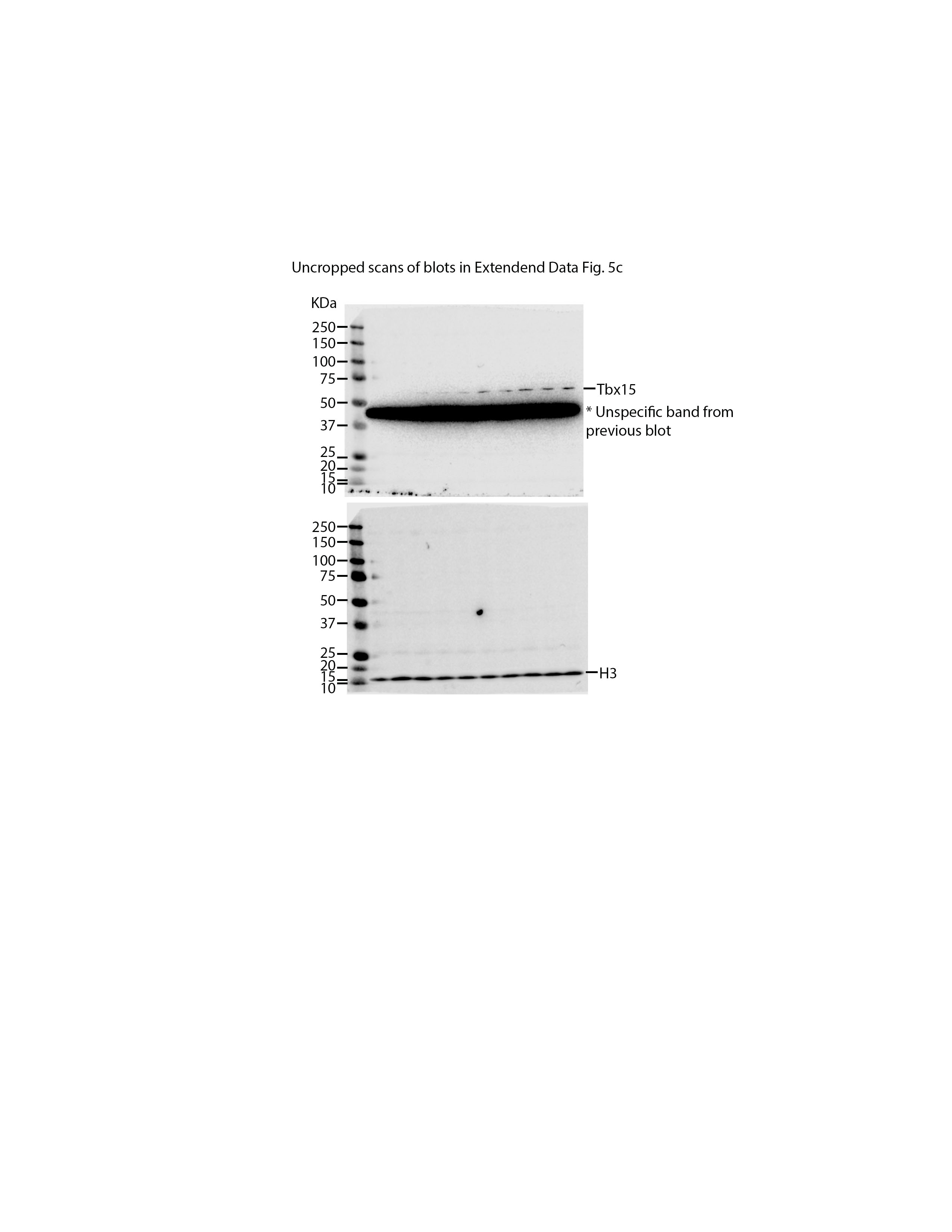

Supplement: Supplementary file 8 — Unprocessed western blots. [file 44161_2023_214_MOESM8_ESM.jpg]

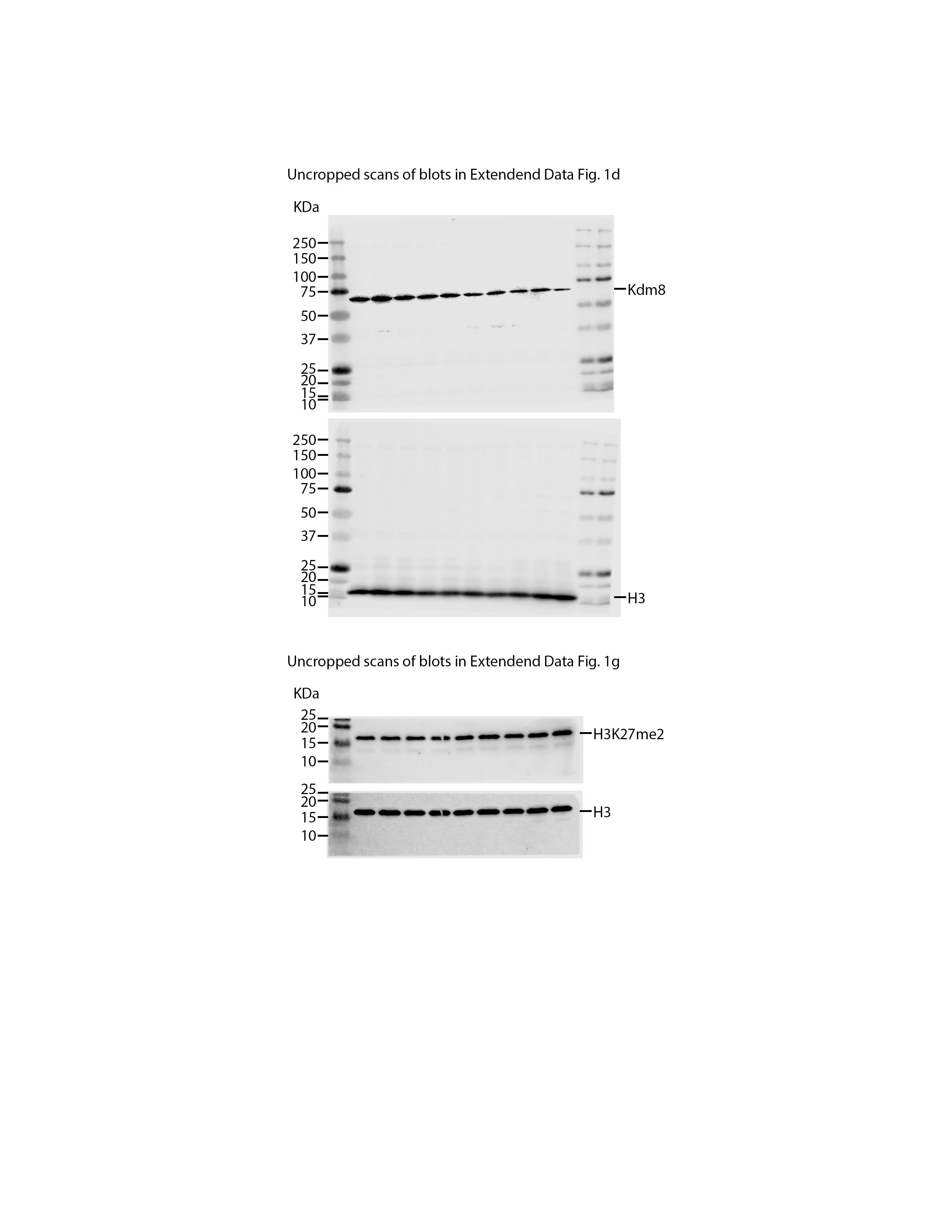

Supplement: Supplementary file 10 — Unprocessed western blots. [file 44161_2023_214_MOESM10_ESM.jpg]

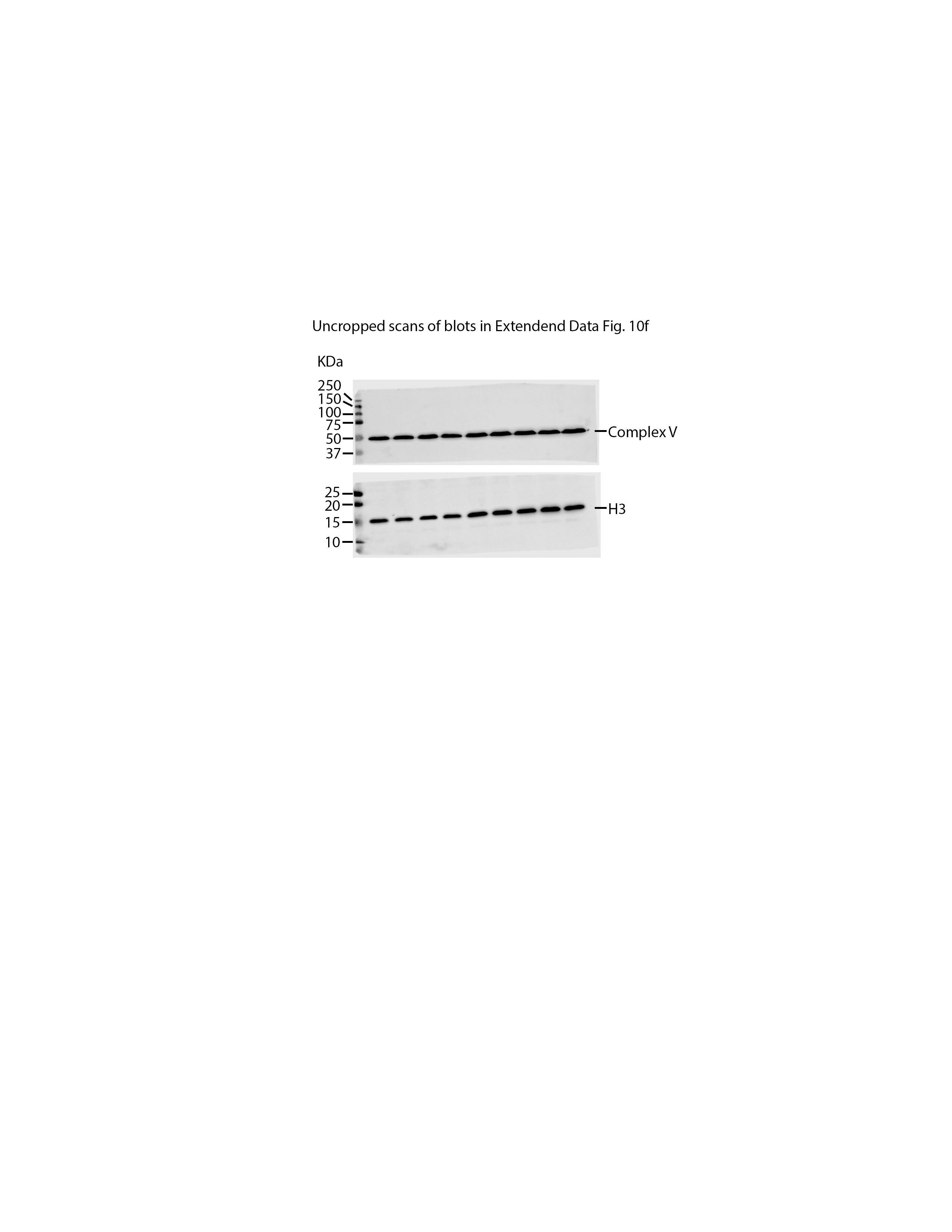

Supplement: Supplementary file 12 — Unprocessed western blots. [file 44161_2023_214_MOESM12_ESM.jpg]
